# *Trans*-cinnamaldehyde-related overproduction of benzoic acid and oxidative stress on *Arabidopsis thaliana*

**DOI:** 10.3389/fpls.2023.1157309

**Published:** 2023-04-21

**Authors:** David López-González, Yolanda Ferradás, Fabrizio Araniti, Elisa Graña, José M. Hermida-Ramón, María Victoria González, Marta Teijeira, Manuel Rey, Manuel J. Reigosa, Adela M. Sánchez-Moreiras

**Affiliations:** ^1^ Departamento de Bioloxía Vexetal e Ciencia do Solo, Facultade de Bioloxía. Universidade de Vigo, Vigo, Spain; ^2^ Instituto de Ciencias de la Vid y del Vino, Consejo Superior de Investigaciones Científicas, Universidad de La Rioja, La Rioja, Spain; ^3^ Departamento de Biología Funcional, Universidade de Santiago de Compostela, Santiago de Compostela, Spain; ^4^ Dipartamento di Science Agrarie e Ambientali – Produzione, Territorio, Agroenergia, Università Statale di Milano, Milano, Spain; ^5^ Departamento de Química Física, Facultade de Química, Universidade de Vigo, Vigo, Spain; ^6^ Departamento de Química Orgánica, Facultade de Química, Universidade de Vigo, Vigo, Spain; ^7^ Instituto de Investigación Sanitaria Galicia Sur, Hospital Álvaro Cunqueiro, Vigo, Spain

**Keywords:** *Arabidopsis*, *trans*-cinnamaldehyde, cinnamic acid, ALDHs, oxidative stress, hormones

## Abstract

**Introduction:**

*Trans*-cinnamaldehyde is a specialised metabolite that naturally occurs in plants of the Lauraceae family. This study focused on the phytotoxic effects of this compound on the morphology and metabolism of *Arabidopsis thaliana* seedlings.

**Material and methods:**

To evaluate the phytotoxicity of *trans*-cinnamaldehyde, a dose-response curve was first performed for the root growth process in order to calculate the reference inhibitory concentrations IC50 and IC80 (*trans*-cinnamaldehyde concentrations inducing a 50% and 80% inhibition, respectively). Subsequently, the structure and ultrastructure of the roots treated with the compound were analysed by light and electron microscopy. Based on these results, the following assays were carried out to in depth study the possible mode of action of the compound: antiauxinic PCIB reversion bioassay, determination of mitochondrial membrane potential, ROS detection, lipid peroxidation content, hormone quantification, *in silico* studies and gene expression of ALDH enzymes.

**Results:**

*Trans*-cinnamaldehyde IC50 and IC80 values were as low as 46 and 87 μM, reducing the root growth and inducing the occurrence of adventitious roots. At the ultrastructural level, the compound caused alterations to the mitochondria, which were confirmed by detection of the mitochondrial membrane potential. The morphology observed after the treatment (i.e., appearance of adventitious roots) suggested a possible hormonal mismatch at the auxin level, which was confirmed after PCIB bioassay and hormone quantification by GC-MS. The addition of the compound caused an increase in benzoic, salicylic and indoleacetic acid content, which was related to the increased gene expression of the aldehyde dehydrogenase enzymes that can drive the conversion of *trans*-cinnamaldehyde to cinnamic acid. Also, an increase of ROS was also observed in treated roots. The enzyme-compound interaction was shown to be stable over time by docking and molecular dynamics assays.

**Discussion:**

The aldehyde dehydrogenases could drive the conversion of *trans*-cinnamaldehyde to cinnamic acid, increasing the levels of benzoic, salicylic and indoleacetic acids and causing the oxidative stress symptoms observed in the treated seedlings. This would result into growth and development inhibition of the *trans*-cinnamaldehyde-treated seedlings and ultimately in their programmed-cell-death.

## 1 Introduction

Weeds are one of the main problems in agriculture, as they cause significant losses in crop yields. For example, in rice crops in Malaysia, weeds can cause yield losses of 5% to 72%, depending on the sowing season, rice variety, number of weeds, etc. (Karim et al., 2004). For this reason, it is important to correctly manage the weeds to obtain a large production of quality crops (Dayan et al., 2009). A significant step forward in controlling weeds was the use of herbicides that replaced the manual work of farmers, who, until then, had to pull out the weeds themselves, thus increasing productivity and reducing costs (Gianessi and Reigner, 2007). Nonetheless, the massive use of herbicides for the last two decades has eventually resulted in the growth of weeds that can resist certain herbicides, apart from the impact that synthetic herbicides have on the ecosystem’s health (Green, 2014; Myers et al., 2016). Furthermore, many herbicides can leach into deep waters, posing a threat to other species (Ueji and Inao, 2001). For this reason, that alternatives to such herbicides are needed, with the goal of finding another cultural practice to control weeds that are more sustainable for the ecosystem and minimise the issue of resistance, as well as the impact on the environment.

The use of natural products is an alternative to using synthetic herbicides. Natural products have a high structural diversity, which makes them excellent candidates for the discovery of new modes of action that differ from the modes of action of synthetic herbicides (Duke et al., 2000). Natural herbicides, or bioherbicides, can derive from secondary metabolites of plant origin and can therefore be involved in plants’ response to stress. Due to their natural origin, they tend to remain in the environment for a short time and to have fewer adverse effects on the rest of the organisms in the ecosystem. Different types of bioherbicides in the market derive majorly from fungi or bacteria, such as Woad Warrior^®^ or Organo-Sol^®^ (Bailey, 2014). However, some bioherbicides already derive from secondary metabolites of plant origin, such as Beloukha^®^, containing pelargonic acid, extracted from rapeseed oil (Cordeau et al., 2016).

Finding natural compounds to be used as herbicides is such a complex task that frequently secondary metabolites with proven biological activity on other organisms, or isolating compounds of plants with known allelopathic properties, are used to find phytotoxic compounds (Dayan et al., 2000).

*Trans*-cinnamaldehyde (*trans*-3-phenyl-2-propenal) is a secondary metabolite of plant origin that belongs to the phenols group. It can be naturally found in plants of the Lauraceae family, particularly in species of the *Cinnamomum* genus, such as *Cinnamomum osmophloeum* and *C. zeylanicum*; it is the main component of the cinnamon essential oil (Peters and Caldwell, 1994). This compound has known fungicidal (Neri et al., 2007), anticancer (Fang et al., 2004), and antimicrobial activities (Helander et al., 1998; Gill and Holley, 2004; Shahverdi et al., 2007), and has protective effects on the cardiovascular system (Rao and Gan, 2014), besides being used as flavour and aroma in many food products such as ice-creams, beverages, chewing gums, etc. (Peters and Caldwell, 1994). It can also prevent the browning process of lettuce after cutting it (Fujita et al., 2006).

Different cinnamon oil-based products with the presence of cinnamaldehyde, can be found for sale. Some examples are the insecticide Biocinn (Farmagro, 2022) or the insecticide and fungicide Seican^®^ (Seipasa, 2023). These pesticides also include a high content of cinnamic acid, which has been found to show different functions such as anti-microbial (Letsididi et al., 2018), anti-cancer (Zhu et al., 2016), anti-obesity (Wang et al., 2020), etc. In addition, this compound can also affect maize roots in different ways depending on the type of root and the stage of development (Lupini et al., 2016), and has been related as well to an increased production of salicylic acid in maize (Araniti et al., 2018b). However, little is known about the mode of action of *trans*-cinnamaldehyde (TC).

Therefore, this study aims to analyse the phytotoxic activity of TC on seedlings of the model species *Arabidopsis thaliana* L., and to elucidate the mechanism/s of action of this compound on plant metabolism. To achieve this objective, the effects of the compound were studied both structural and ultra-structurally, as well as its effects on hormonal balance and the expression of genes involved in TC metabolism, selected from the results obtained through molecular docking.

## 2 Materials and methods

### 2.1 Dose-response curve

*Arabidopsis thaliana* (L.) Heynh seeds of the Columbia ecotype (Col-0) were sterilised with 50% EtOH and 0.5% Triton X-100 NaOCl for 3 minutes each. Secondly, three 1-minute washes in autoclaved distilled water were performed. Lastly, the seeds were soaked in 0.1% agar at 4 °C for 3 days to promote their stratification. After that, seeds were sown in square Petri dishes (100 x 100 x 15 mm) in autoclaved plant agar medium (pH 6.0) (Duchefa, Haarlem, Netherlands) with a mixture of macro and micronutrients (Murashige-Skoog, Sigma-Aldrich, St. Louis USA) (Murashige and Skoog, 1962), supplemented with 1% sucrose. Each Petri dish contained approximately 35 mL of medium. *Trans*-cinnamaldehyde (*trans*-3-phenyl-2-propenal, TC, Sigma Aldrich) was diluted in the medium at different concentrations (0, 50, 100, 200, 400, 800, and 1200 µM) using 0.1% pure EtOH as solvent. Control dishes also had 0.1% pure EtOH. A total of 24 seeds were sown in each dish, and 5 dishes were prepared per concentration. After sowing the seeds, the Petri-dishes were vertically placed in a growth chamber at 22 ± 2 °C and relative humidity of 55%. The seeds were grown using a short-day photoperiod with 8 h light (120 µmol m^−2^ s^−1^) and 16 h darkness to promote germination. The seeds were under these conditions for 14 days. After this period, the number of germinated seeds was counted, and the length of each seedling was measured, calculating the average root length for each treatment. The germination and root growth values were expressed as a percentage of the control and were used to determine the IC_50_, IC_80_ and LCIC values (concentrations of the compound that cause a 50%, 80% and 100% inhibition, respectively). The dishes were scanned with an ImageScanner III, using the EPSON EXPRESSION 10000 XL software.

A morphological analysis was also conducted (root thickness, presence or absence of lateral roots and root hairs, growth direction, torsion effects, etc.) using a Nikon SMZ 1500 stereo microscope (Melville, NY, USA), for a detailed study of the compound’s effects on seedlings’ morphology.

The data obtained were analysed with the statistical program SPSS v.15.0. A total of 120 seedlings were measured for each of the 7 treatments. First, the data were analysed by creating a box plot to detect outliers. The normality of the data was tested by a Kolmogorov-Smirnov test, followed by a Levene’s test to assess the homoscedasticity of the data. When the data were homoscedastic, an ANOVA test with DMS was used, whereas when the data were not homoscedastic, they were analysed with the Tamhane T2 test. A Kruskal-Wallis test was used to analyse non-normal data.

Different models for curvilinear regressions were used to adjust the dose-response curve, selecting the one with the best adjustment according to the correlation coefficient (R2).

### 2.2 Structural and ultrastructural root analysis

The root apex of 40 *Arabidopsis thaliana* seedlings grown for 7 or 14 days on IC_0_ and IC_50_ TC concentrations were cut (1–2 mm), soaked immediately in 0.1 M cacodylate buffer (pH 7.2), with the fixative glutaraldehyde (5%), and kept at 4 °C for 4 h. Subsequently, they were washed 3 times, every 4 h, using freshly prepared 0.1 M cacodylate buffer (pH 7.2). After washing, the samples were soaked in 0.1 M cacodylate buffer with 2% OsO_4_ for 3 h and then in 10% acetone solution with 2% uranyl acetate for 1 h. The samples were dehydrated by soaking the roots in acetone solutions with increasing concentrations for different periods, and then included in 100% Spurr resin at 4 °C. After the inclusion, the samples were placed in pure resin moulds to be polymerised for 2–3 days at 60 °C. Then, semi-thin (0.7 µm) and ultrathin (50–70 nm) sections were cut for light and electron microscopy, respectively. Semithin sections were stained with toluidine blue to conduct a structural analysis of the roots with a Nikon Eclipse 800 light microscope, equipped with a Sight Nikon DS-U2 digital camera, and the NIS-Elements D 2.30 SP1 software. Ultrathin sections were contrasted with uranyl acetate (2%) for 30 min and lead citrate for 12 min (Reynolds, 1963). The samples were washed with autoclaved distilled water for 2 min. The sections were picked up with copper grids, 100 and 200 mesh, and observed with a high-contrast transmission electron microscopy JEOL JEM-1010 (80 kv), equipped with a CCD Orius and Digital Montage Plug-in camera (Gatan Inc., Gatan, CA, USA), and the Gatan Digital Micrograph software (Gatan Inc.).

### 2.3 Anti-auxin bioassays

A bioassay with the antiauxin *p*-chlorophenoxyisobutyric acid (PCIB) (Sigma Aldrich) was conducted on *A. thaliana* seedlings treated with and without *trans*-cinnamaldehyde. For that, two PCIB concentrations (15 and 30 μM) (Oono et al., 2003; Biswas et al., 2007; Tamás et al., 2012), and the IC_0_, IC_50_ and IC_80_ *trans*-cinnamaldehyde were used in the bioassay for single treatments (15PCIB, 30PCIB, IC_0_TC, IC_50_TC, IC_80_TC), but also a combination of both compounds in different concentrations (15PCIB/IC_50_TC, 30PCIB/IC_50_TC, 15PCIB/IC_80_TC, and 30PCIB/IC_80_TC) were conducted in this bioassay. A total of 5 replicates were used for each of the 9 treatments. The sowing method was the same as the method used for the previously mentioned germination and growth bioassays.

After 14 days of growth, the dishes were observed and photographed under the stereo microscope (Nikon SMZ 1500).

### 2.4 Mitochondrial membrane potential

The mitochondrial membrane potential (ΔΨm) was measured using the fluorochrome 5,5′,6,6′-tetrachloro-1,1′,3,3′-tetramethyl-benzimidazol carbocyanine iodine (JC-1; Invitrogen, UK), according to Díaz-Tielas et al. (2012). The fluorochrome is a lipophilic cation that selectively enters mitochondria when the mitochondrial potential is high, forming J-aggregates (a concentration-dependent fluorescent nematic phase), which emit red fluorescence. On the contrary, when the mitochondrial potential is depolarised, JC-1 forms monomers in the cytoplasm that emit green fluorescence, as the fluorochrome cannot go through the mitochondrial membrane (Smiley et al., 1991).

The ΔΨm was measured in control plants (IC_0_ TC) and plants treated with IC_50_ and IC_80_ TC, using a solution of 10 μg mL^−1^ JC-1 in a buffer of 10 mM HEPES (pH 7.2), 1 mM CaCl_2_, 1 mM MgCl_2_ and 0.6 mM sorbitol. The control seedlings and those treated with TC after 7 and 14 days were permeabilised in 5% DMSO for 1 h and incubated in the dark in the JC-1 solution for 30 to 40 min. The samples were then washed twice with the buffer to remove excess fluorochrome. Valinomycin (Invitrogen, Molecular Probes), which depolarises the mitochondrial membrane, was used as a positive control. For this positive control, the seedlings were incubated in 0.5 μg mL^−1^ valinomycin for 30 min before incubation with JC-1.

The roots were observed and photographed with the Leica TCS SP5 confocal microscope (Wetzlar, Germany), with a 63x immersion objective, a 488 nm excitation wavelength (argon laser), and a 535 nm emission wavelength for the green fluorescence of JC-1 monomers and 590 nm emission wavelength for the red fluorescence of J-aggregates, using a hybrid detector.

### 2.5 Superoxide determination

The *in situ* determination of the superoxide anion (O_2_^−^) was carried out following the protocol of Wang and Zou (2018), with some modifications. Seven and 14 days-old control and IC_50_ and IC_80_ TC-treated seedlings were incubated in the dark at room temperature in a 5 µM dihydroethidium (DHE) staining solution for 15 min. Subsequently, the seedlings were washed twice in deionised water and placed on a glass slide to be immediately visualised in a fluorescence microscope with red and green excitation filters. The images were then analysed with ImageJ software (National Institutes of Health, USA), and the corrected total cell fluorescence (CTCF) was calculated from the equation:

CTCF = Integrated Density − (Area of selected cell × Mean fluorescence of background readings)

Superoxide quantification data (3 replicates per treatment) were analysed with the statistical program SPSS v.15.0 using a t test (*p* ≤ 0.05) (N=3).

### 2.6 Hydrogen peroxide detection

Control and treated seedlings with IC_50_ and IC_80_ TC for 7 and 14 days were incubated in the dark in 1 mg mL^−1^ 3,3-diaminobenzidine (DAB) in acidified water (pH 3.8) for 8 h. After incubation, the DAB was removed, and the seedlings were left under the light for 5 min. Subsequently, seedlings were washed three times with 50% EtOH. Then, the seedlings were preserved in 70% glycerol, and their radicles were observed with a Nikon Eclipse 800 light microscope equipped with a Sight Nikon DS-U2 digital camera and the NIS-Elements D 2.30 SP1 software. Images were analysed with the Image ProPlus software (Media Cybernetic Inc., Bethesda, MD, USA), and the integrated optical density (IOD) of the DAB-stained roots was determined. DAB quantification data, i.e., IODs, were analysed in 3 replicates per treatment with the statistical program SPSS v.15.0 with a t test (*p* ≤ 0.05) (N=3).

### 2.7 Lipid peroxidation

Lipid peroxidation was indirectly determined by measuring the content of malondialdehyde (MDA), according to Hodges et al. (1999), with some modifications. 100 mg of 14 days control and IC_50_ TC treated seedlings were powdered in liquid nitrogen and homogenised with 1 mL of 80% ethanol and then centrifuged at 3000 rpm at 4 °C for 10 min. The supernatant was then incubated for 25 min at 95˚C with 20% TCA containing 0.01% hydroxytoluenebutylate with and without 0.5% thiobarbituric acid (TBA). The reaction was then stopped in ice. Finally, samples were centrifuged at 3000 rpm at 15 °C for 10 min, and the absorbance of the supernatant was measured at 440, 532 and 600 nm. MDA equivalents were calculated using the following equations:


$$A=[(ABS_{532+TBA})-(ABS_{600+TBA})]-[(ABS_{532-TBA})-ABS_{600-TBA})]$$

$$B=[(ABS_{440+TBA-}ABS_{600+TBA})\times 0,0571]$$

$$MDA\;equivalents\;(nmol\;mL^{-1})=(A-B/157.000)\times 10^{6}$$

The data obtained were analysed with the statistical program SPSS v.15.0. First, the data were analysed by creating a box plot to detect outliers. The normality of the data was tested by a Kolmogorov-Smirnov test, followed by a Levene’s test to assess the homoscedasticity of the data. When the data were homoscedastic, an ANOVA test with DMS was used, whereas when the data were not homoscedastic, they were analysed with the Tamhane T2 test. A Kruskal-Wallis test was used to analyse non-normal data (N=6).

### 2.8 Benzoic, salicylic and indoleacetic acid quantification

#### 2.8.1 Sample extraction and derivatisation

Fourteen days control and IC_50_ TC-treated seedlings were frozen in liquid N_2_ and then extracted according to Villas-Bôas et al. (2003), with some modifications. An amount of 20 µL of 20 mg mL^−1^ indole propionic acid, which was used as an internal standard, was added to the pulverised samples. Subsequently, 200 µL NaOH (1% w/v), 147 µL MeOH and 34 µL pyridine were added to the samples and mixed in the vortex for 40 s. Then, 400 µL chloroform were added to the samples and shaken for 10 s. Lastly, 400 µL of 50 mM NaHCO_3_ were added. Everything was shaken for 20 s and centrifuged at 16.11 x *g* for 1 min. The organic fraction obtained was transferred to new 2 mL centrifuge tubes, whereas the aqueous fraction was removed using Na_2_SO_4_ anhydrous. An aliquot of 1 mL was used for GC-MS analysis.

#### 2.8.2 GC-MS analysis

The GC-MS analysis was conducted using a Thermo Fisher gas chromatograph (Trace 1310) with a single quadrupole mass spectrometer (ISQL). A TG-5MS 30 m x 0.25 mm x 0.25 µm capillary column (Thermo Fisher Scientific, Waltham, USA) was used, and helium was the carrier gas selected, with a flow rate of 1 mL min^−1^. The injector and the transfer line were set at 250 °C and 270 °C, respectively. Three microliters of the sample were injected at a pulse pressure of 35 psi for 1 min. The following temperature was set: isocratic at 40 °C for 1 min, from 40 °C to 320 °C at a ratio of 20 °C per minute, and isocratic at 320 °C for 2 minutes. The ion source was set at 200 °C, and the solvent delay was 4.5 min. The mass spectrum was recorded in electron impact form (EI) at 70 eV, scanning at 50–400 m/z to select the adequate EI mass fragments for each analyte. The MS mode applied was the selected ion monitoring (SIM), using a quantifier ion (m/z) and two qualifier ions (m/z) for each molecule; in particular: benzoic acid, Me ester (105, 136, 77); 3-indole acetic acid, Me ester (189, 103, 77); salicylic acid, Me ester (120, 152, 92). The compounds were identified by comparing the molecules’ mass spectra and relative retention time to derive authentic standards using commercial libraries (i.e., NIST 2005 and Wiley 7.0).

A completely randomized design was applied with 3 replicates. Data were evaluated for normality with the Kolmogorov-Smirnov test and tested for homogeneity of variances with Levene’s test. The statistical significance of differences among group means was estimated by Student t-test (*p* ≤ 0.05). Data were expressed as a percentage of the control.

### 2.9 Homology models

The full-length residue sequence of aldehyde dehydrogenase family 2 member B4 from *Arabidopsis thaliana* (ALDH2B4_ARATH) was imported from the Universal Protein Resource, UniProt (http://www.uniprot.org, code: Q9SU63). Its three-dimensional (3D) structure was developed by homology modelling using the crystal structure of aldehyde dehydrogenase family 2 from *Zea mays* (ZmALDH2), imported from Protein Data Bank server (PDB, https://www.rcsb.org/; ID: 4PXL; 2.25 Å resolution), as a template.

The ZmALDH2 protein was prepared and converted to an ICM object using Molsoft ICM suite (Abagyan and Totrov, 1994), and its residues sequence was extracted.

Both protein sequences were aligned using the built-in Molsoft ICM (Molsoft LLC, version 3.9-2e) alignment tool. Finally, the result of the sequence alignment was used for homology modelling. With full refinement, the homology modelling was performed using Molsoft’s ICM Homology tool. Ramachandran plots and ICM Protein Health tool were used to check the quality of the refinement. ICM-Molsoft protein health option calculates the energy strain of a macromolecule and allows optimisation procedure to rectify miscalculations when necessary.

### 2.10 Molecular docking models

#### 2.10.1 Ligand preparation

*Trans*-cinnamaldehyde structure (PubChem CID 637511) was converted into ICM object using Molsoft ICM suite. In this step, 2D structure was converted to 3D structure, and all conformations were calculated.

#### 2.10.2 Docking poses

The ligand was docked into the predicted Homology model using ICM flexible docking method according to their most energetically favourable protein binding conformation (Abagyan and Totrov, 1994). ICM uses a scoring function based on the docked binding affinities of protein-ligand complexes. Low scores suggest better affinities. The best poses were selected according to their scoring function values.

### 2.11 Molecular dynamics simulations

The starting configuration of the TC-Homol_ALDH2B4 complex was obtained from the docking model.

For the complex calculation, Tian et al. (ff19SB) force field was employed for the Homol_ALDH2B4 (Tian et al., 2020), and for the TC the generalised AMBER force field (gaff) parameters were used. The TC-Homol_ALDH2B4 complex was neutralised by the addition of calcium and sodium ions and solvated with a TIP3P water truncated octahedron box at 14 Å from solute surface. Before MD simulations, the complex was minimised without restraints with the conjugate gradient method at 0 K using periodic boundary conditions in a NVT ensemble. Long-range electrostatic interactions were calculated based on the particle mesh Ewald method with a cut-off value of 12 Å or the following simulations. After minimisation, the system was subjected to a MD simulation for 15 ns to gradually increase the temperature from 0 K to 300 K in a NVT ensemble. The time-step of this simulation was 1 fs, and a Langevin thermostat collision frequency of 1.0 was used to control the temperature. The SHAKE algorithm was applied to constrain all bond interactions involving hydrogen. The Langevin thermostat and SHAKE algorithm were also used in the following MD equilibration of the system. This simulation was performed for 40 ns in a NPT ensemble with a time-step of 2 fs. The pressure was maintained at 1 atm using the Berendsen barostat with a relaxation time of 2 ps. The same technical specifications of the equilibration were used in the production MD simulation that generated the conformations employed to perform the analysis. Thus, after the equilibration, the system was subjected to a MD simulation for 200 ns and the trajectory was stored every 2 ps. Stored configurations were analysed using the cppTraj module (Roe and Cheatham, 2013) included in AmberTools. All MD simulations were performed using the AMBER package (Case et al., 2022), and VMD (Humphrey et al., 1996) to visualise structures and trajectories resulting from the MD simulations.

### 2.12 Total RNA extraction and cDNA synthesis

Three pools (biological replicates) from roots or shoots (about 60 mg fresh weight) of at least 200 frozen seedlings grown for 14 days in a growth medium with IC_0_ or IC_50_ *trans*-cinnamaldehyde were ground to a fine powder in liquid nitrogen using a Mikro-Dismembrator-S (Sartorius AG, Goettingen, Germany). RNA was extracted using the SV Total RNA Isolation System Kit (Promega, Madison, WI, USA) according to the manufacturer’s instructions with some modifications. PVP (1%) was added to the RNA lysis buffer and extracts were incubated with DNAse for 60 min and with nuclease-free water for 1–2 min prior to centrifugation.

The RNA concentration and absorbance ratios (260/280 nm and 260/230 nm) were determined using a NanoDrop 2000c Spectrophotometer (Thermo Fisher Scientific Inc., Waltham, MA, USA). RNA integrity and concentration were confirmed using an Agilent 2100 bioanalyser (Agilent, Mississauga, ON, Canada). First-strand cDNA was synthesised from 0.5 µg of total RNA using the iScript cDNA Synthesis Kit (Bio-Rad, Hercules, CA, USA), according to the manufacturer’s instructions.

### 2.13 Primers design, real-time qPCR and data analysis

qPCR primers (**Table 1**) were designed using the NCBI primer design tool. The accurate amplifications in the genome of *A. thaliana* were verified by conventional PCR and Sanger sequencing. *AT2G28390* (SAND family), *AT5G08290* (Mitosis protein YLS8) and *AT5G15710* (F-box protein) were used as reference genes (Remans et al., 2008) for relative expression normalisation. Roots or shoots from plantlets germinated in a medium without *trans*-cinnamaldehyde were considered as the calibrator group. Three biological samples were included per treatment, and each sample was tested in duplicate.

**Table 1:** Nucleotide sequence, amplicon size and qPCR efficiency of the oligonucleotide primers designed for this study.

Gene name	Gene ID	Primers sequence (forward/reverse)	Amplicon size (bp)	qPCR efficiency
*ALDH2B4*	*AT3G48000*	GGCGAAGTCATTGCTCATGT/TGCCATTGTCCCATGTCTCT	199	2.001
*ALDH2B7*	*AT1G23800*	CATGAGGCTGGACTTCCTGA/ACCCGGTGAAAGCAACCTTA	112	2.000
*ALDH2C4*	*AT3G24503*	TGATATTGACAAAGCCGCCG/AGGATCACCAACGGTCCAAT	160	2.001
*ALDH3F1*	*AT4G36250*	TCGCGCCTACTCTGATTGAT/AGACGATGGAAGCTTGGACA	159	2.001
*ALDH3H1*	*AT1G44170*	TTTGGCCCTCTCCTTCCAAT/TGACTACTATGCCTCCAGCG	160	1.995
*ALDH3I1*	*AT4G34240*	TCACTGGTGGAGCAAGAGTT/GATCCTCCTAGCAGCCACTT	140	1.995
*ALDH7B4*	*AT1G54100*	TCAGAACGCCCTAACCACAT/CACCACACATGGCCGTAAAA	250	2.001
*ALDH11A3*	*AT2G24270*	CCGATAGCTTCCCCGGTAAT/GGAGGTTTGAGGACAAGGGA	163	1.995
*ALDH22A1*	*AT3G66658*	CTTTCTGAGGGCGAACGATG/CACCAAGGGGATGGAACTCT	97	2.000

Gene expression analyses were performed following the Minimum Information for publication of Quantitative real-time PCR Experiments (MIQE) guidelines (Bustin et al., 2009). The qPCR reactions were carried out using 1X SsoFast™ EvaGreen^®^ Supermix (Bio-Rad) following manufacturer’s instructions, with 0.4 µM of each primer and 2 µL of diluted cDNA (1:10). The reactions were performed in 96-well plates in an iCycler iQ™ real-time thermal cycler (Bio-Rad) as follows: 1 min at 98°C, followed by 40 cycles of 5 s at 98°C and 20 s at 59°C for annealing and extension. Duplicate no-template controls were included in the experiment. Melting curve analysis was performed for all PCR products to confirm the occurrence of specific amplification products and the absence of primer dimer formation (data not shown).

The raw fluorescence data were analysed using LinRegPCR software (Ruijter et al., 2009) to obtain the quantification cycle (Cq) values and the mean PCR efficiency for each primer pair. Relative gene expression was determined and statistically analysed (*p*< 0.05) using the REST-2009^®^ (Qiagen, Pfaffl et al., 2002) with PCR efficiency correction and normalisation by three reference genes and compared with the 2-ΔΔCq method. The Cq values were analyzed using the REST software that allows normalization with one or more reference genes using the ΔΔCq method and simultaneously performs the statistical analysis. The statistical analysis that the REST software performs is a P(H1) test that represents the probability of the alternate hypothesis that the difference between the sample and the control group is because of chance only. The hypothesis test performs 10,000 random reallocations of samples and controls between the two groups and counts the number of times the relative expression on the randomly assigned group is greater than the sample data. This statistical analysis is considered significant at *p*< 0.05.

## 3 Results

Dose-response curves for germination and root and shoot growth demonstrated the phytotoxic potential of *trans*-cinnamaldehyde on the model species *A. thaliana*. Germination was only affected (**Supporting information Figure S1A**) by the highest TC doses tested (800 and 1200 µM), whereas it had a more significant impact on root growth (**Supporting information Figure S1B**), which was strongly affecting by TC at low concentrations, pointing out low values of IC_50_ (46 µM) and IC_80_ (87 µM).

**Figure 1:**
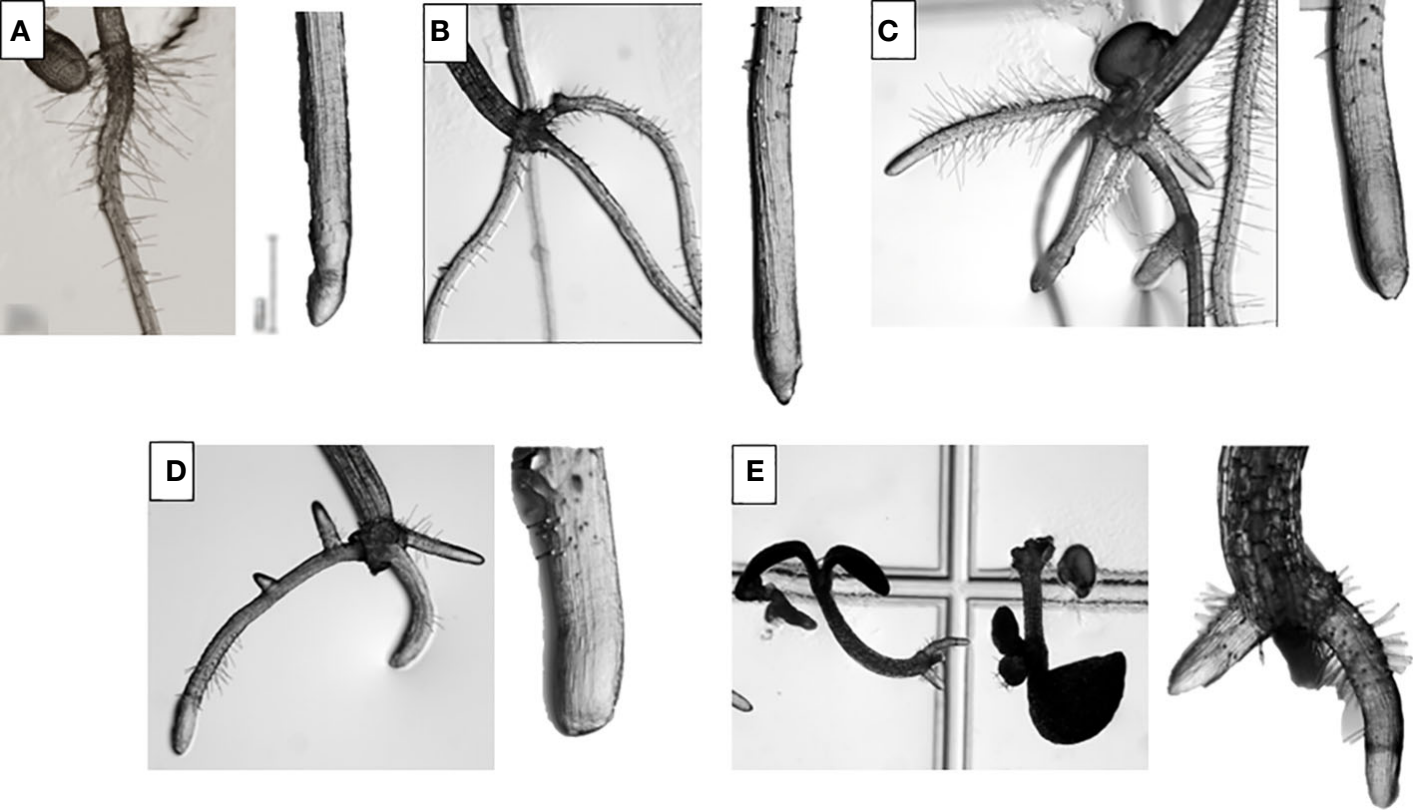
*Arabidopsis thaliana* roots under magnifier after 14 days of growth treated with 0 **(A)**, 50 **(B)**, 100 **(C)**, 200 **(D)** and 400 µM **(E)** of *trans*-cinnamaldehyde.

Regarding morphological analysis (**Figure 1**), TC-treated showed a completely different morphology from the control seedlings. The morphology of the controls was characterised by the presence of a single main root, with root hairs close to the hypocotyl and without adventitious roots. However, in the treated seedlings, adventitious and lateral roots appeared at low TC concentrations (50 and 100 µM) and at higher concentrations (200 and 400 µM) ectopic root hairs appeared close to the root apex. In addition, as the TC concentration increased, the reduction in root length became more evident.

**Figure 2:**
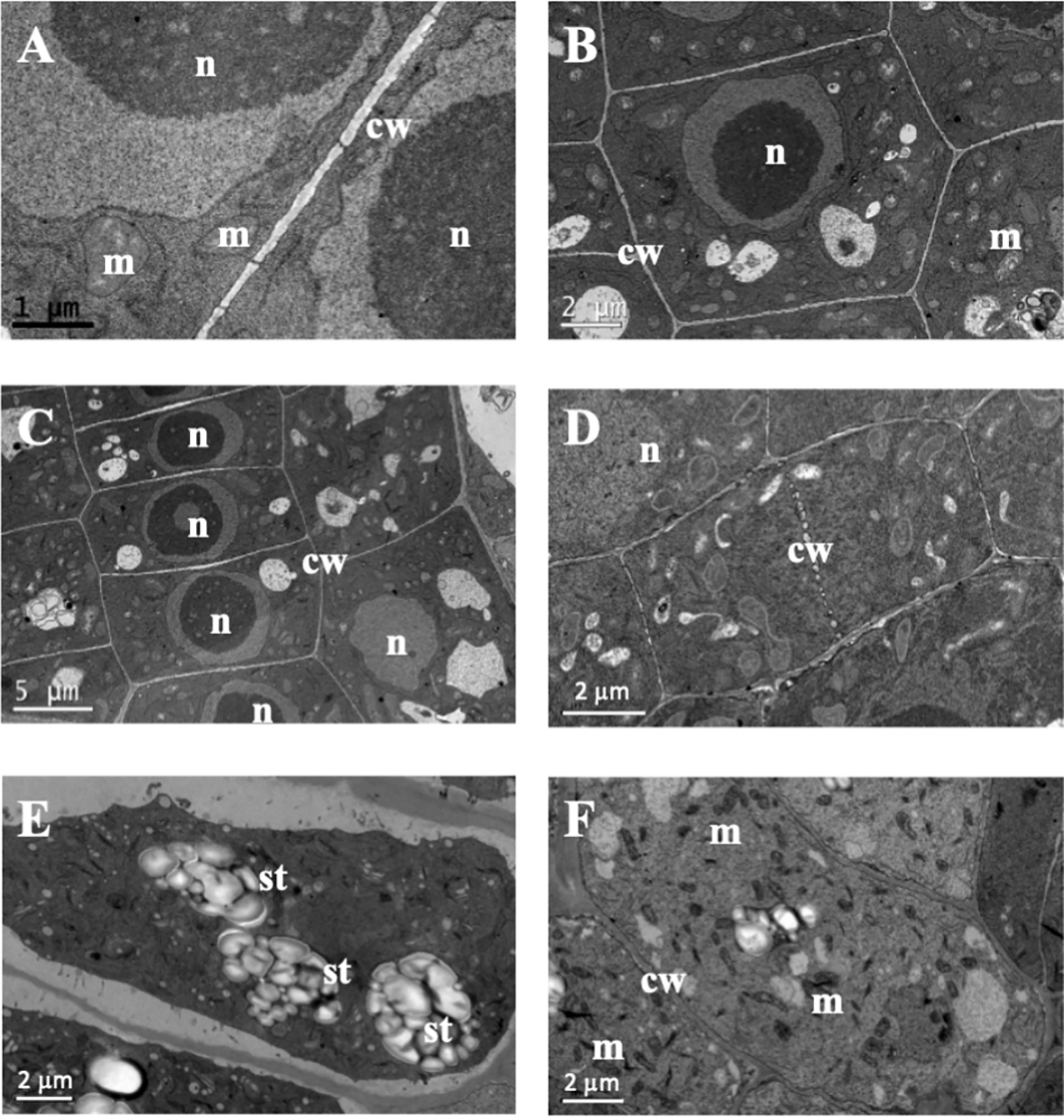
Images of transmission electron microscopy (TEM) of control *Arabidopsis thaliana* roots. **(A)** Straight cell wall separating two cells. **(B, C)** Symmetric control cells with spheric nuclei and regular cell walls. **(D)** Plant cell finishing dividing. **(E)** Statoliths in root cell. **(F)** Control cell with some mitochondria. (n, nucleus; m, mitochondria; cw, cell wall; st, statoliths). Scale bars: **(A)** 1 µm; **(B–E)** and **(F)** 2 µm; **(C)** 5 µm.

**Figure 3:**
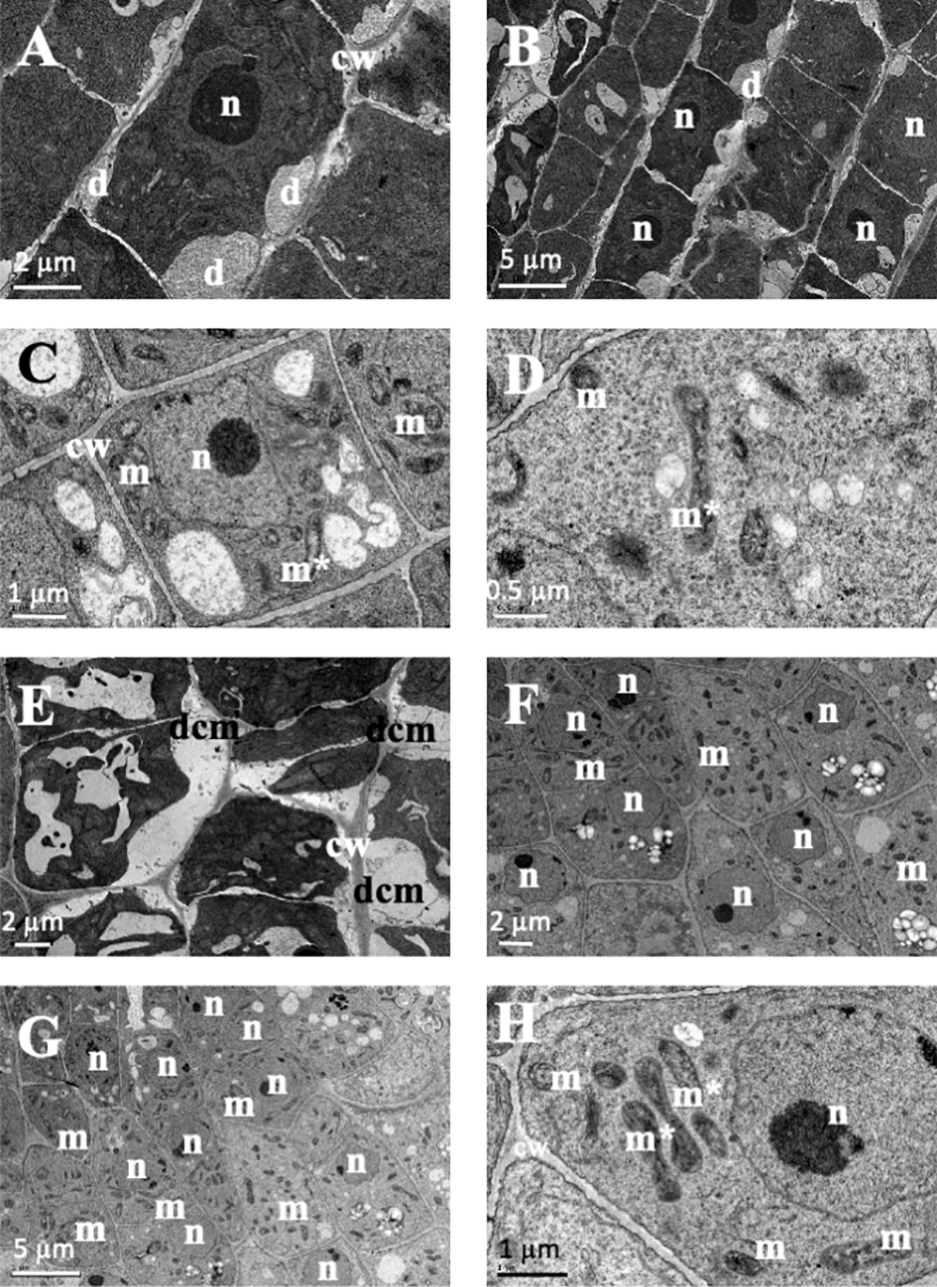
Images of transmission electron microscopy (TEM) of *Arabidopsis thaliana* roots treated with IC_50_ of TC for 7 days. **(A, B)** Cells with deposits between the membrane and wall. **(C, D, H)** Cells with dividing mitochondria. **(E)** Cell with a plasma membrane separated from the cell wall. **(F, G)** Cells with many mitochondria. (n, nucleus; m, mitochondria; cw, cell wall; dcm, detached cell membrane; m*, dividing mitochondria). Scale bars: **(A, E, F)** 2 µm; **(B, G)** 5 µm; **(C, H)** 1 µm; D 0.5 µm.

**Figure 4:**
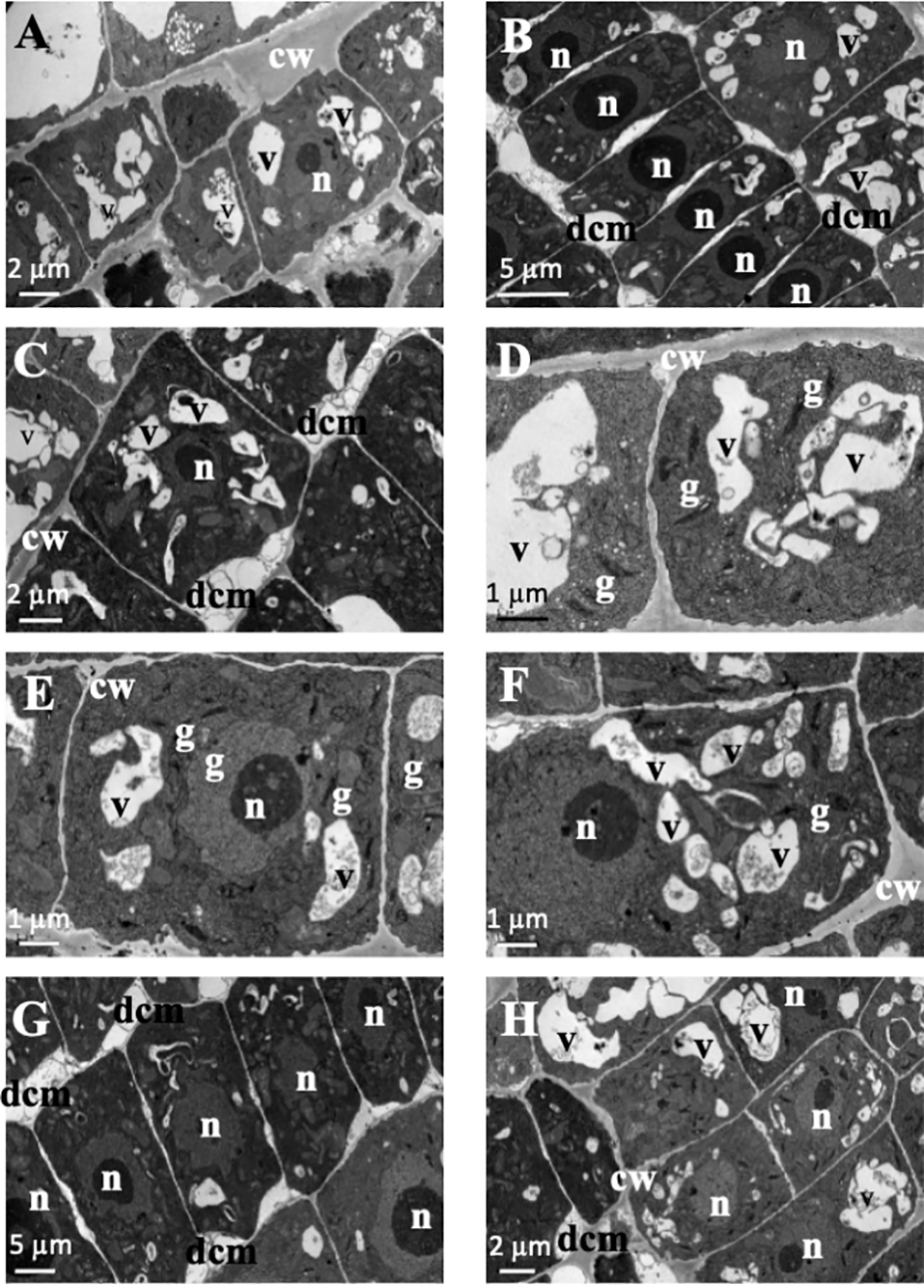
Images of transmission electron microscopy (TEM) of *Arabidopsis thaliana* roots treated with IC_50_ of TC for 14 days. **(A–C, G, H)** Cells with detached cell membranes and cytoplasm vacuolated. **(D–F)** Vacuolated cells with active Golgi apparatus. (n, nucleus; cw, cell wall; dcm, detached cell membrane; v, vacuole; g, Golgi apparatus). Scale bars: **(A, C, H)**: 2 µm; **(B, G)**: 5 µm; **(D–F)**: 1 µm.

In the structural root analysis, the longitudinal semithin sections of *A. thaliana* root apices showed an effect of TC on cellular morphology and organization (**Supporting information Figure S2**). While control seedlings showed uniform-sized cells arranged into uniform rows that constituted the distinct layers of the root, IC_50_ TC-treated roots showed a disorganisation of the calyptra, next to the meristematic zone, characterised by wider cells than those of control roots and with altered division planes. Such effects were more visible after 14 d of IC_50_ treatment (**Figure S2**). Similarly, the ultrastructural analysis conducted in ultra-thin sections with a transmission electron microscope (TEM analyses) revealed clear differences between control and TC-treated seedlings and different effects according to the treatment time. As observed in **Figure 2**, control root images showed cells with a spherical nucleus, decondensed chromatin (**Figures 2B, C**), well-defined and homogeneous cell walls (**Figures 2A, F**), statoliths in the most apical cells (**Figure 2E**) and an amount and disposition of the different organelles that corresponded to a healthy cell. These cells were arranged in rows (**Figure 2C**), showing a correct division pattern (**Figure 2D**). However, 7 days of IC_50_ TC treatment (**Figure 3**) resulted in clear differences when compared to the control. The plasma membrane of TC-treated cells was detached from the cell wall (**Figures 3A, E**), creating a space where several accumulated deposits could also be found (**Figure 3B**). Moreover, cell walls were thicker compared to the control and did not keep the classical shape of the control cells (**Figures 3B, E**). Furthermore, a higher number of mitochondria was also observed compared to the control (**Figures 3F, G**). Many of them were undergoing a division process (**Figures 3C, D, H**). When plants were treated with IC_50_ TC for 14 days (**Figure 4**), we could observe similar TC effects as those observed after 7 days, i.e., thick cell walls, plasma membranes detached form the cell wall, and higher number of mitochondria (**Figures 4A–C, G**). However, other new effects after TC treatment could also be observed, such as the appearance of numerous different-sized vacuoles in the cytoplasm (**Figures 4D, H**), and an increasing number of Golgi apparatus (**Figures 4D–F**), which were very active as attested by the high number of small vesicles found.

**Figure 5:**
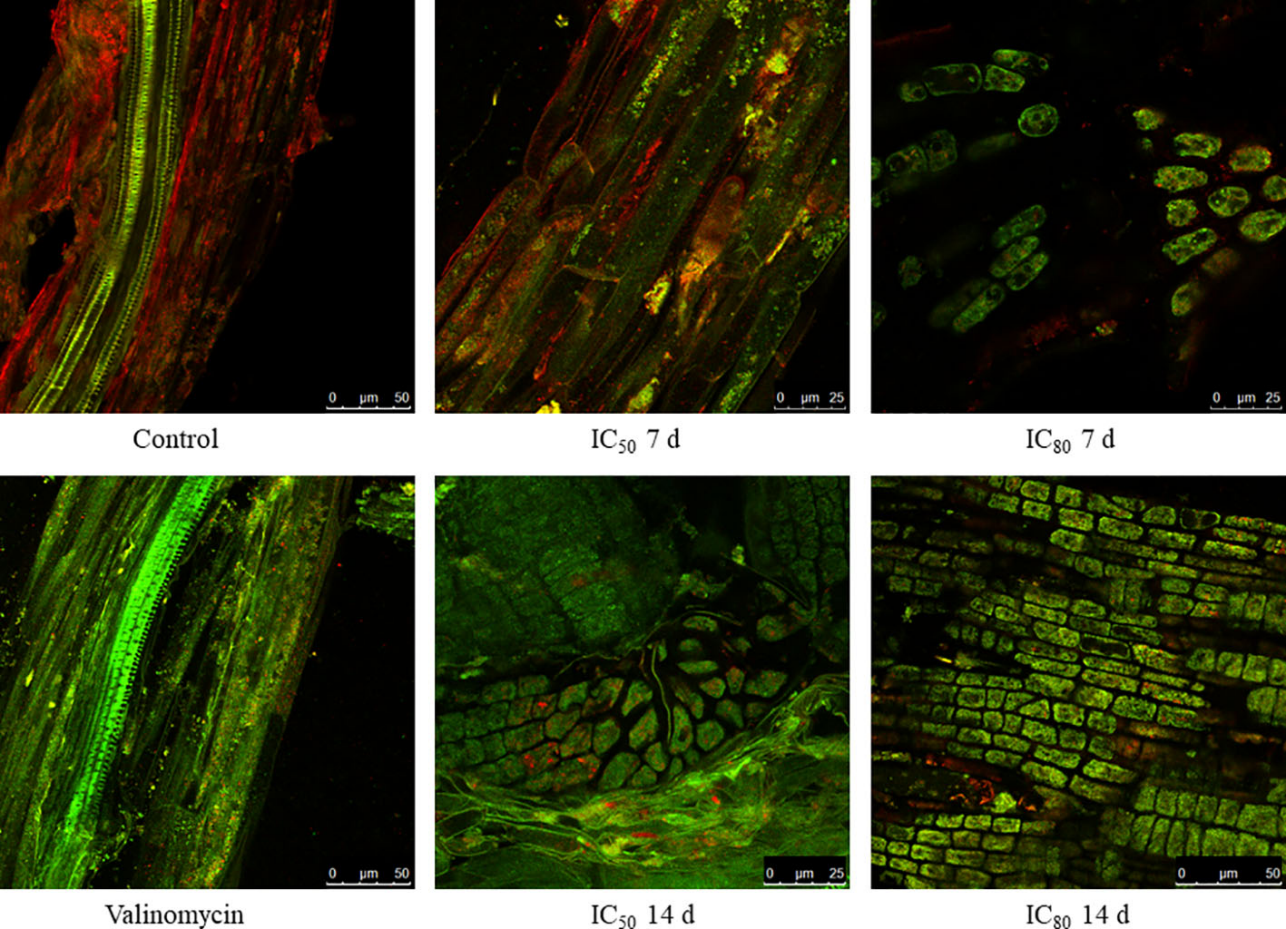
Confocal microscopy images of JC-1 stained *Arabidopsis* roots after treatment with IC_0_ TC (control), IC_50_ TC, and IC_80_ TC for 7 and 14 days. Valinomycin-treated roots are shown as positive control.

Due to the increased number of mitochondria in the cells treated with TC, the fluorophore JC-1 was used to detect possible alterations in the mitochondrial membrane potential (ΔΨm). As observed in **Figure 5**, the cells of control seedling roots emitted red fluorescence, suggesting a correct polarisation of their mitochondrial membrane. In contrast, the cells treated with valinomycin (positive control), a known disruptor of the mitochondrial membrane (Shinohara et al., 2002), showed green fluorescence, indicating that the membrane potential has been altered and JC-1 could not enter the mitochondria. Regarding the seedlings treated with IC_50_ TC for 7 days, most of the fluorescence emitted was red fluorescence as in the control, with a slight increase in green fluorescence. However, those cells treated with IC_80_ TC for 7 days emitted mostly green fluorescence, as found for the positive control, suggesting a depolarisation of the mitochondrial membrane. When cells treated with IC_50_ and IC_80_ TC for 14 days were analysed, a clear alteration of the membrane potential was observed for both treatments, as revealed by the high amount of green fluorescence emitted.

**Figure 6:**
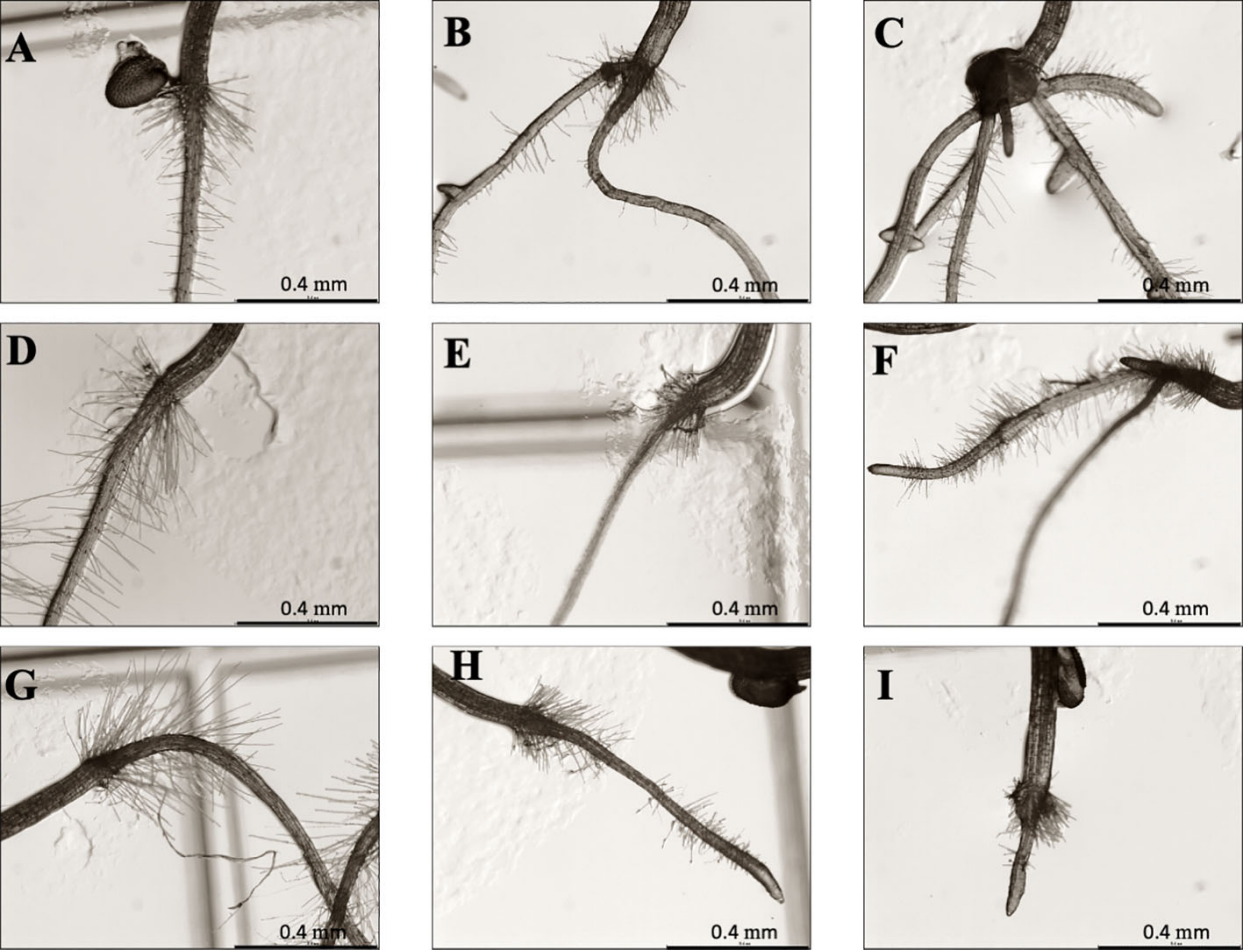
*Arabidopsis* roots after 14 d of PCIB bioassay. **(A)** Control, **(B)** IC_50_ TC, **(C)** IC_80_ TC, **(D)** 15 μM PCIB, **(E)** 15 μM PCIB + IC_50_ TC, **(F)** 15 μM PCIB + IC_80_ TC, **(G)** 30 μM PCIB, **(H)** 30 μM PCIB + IC_50_ TC, **(I)** 30 μM PCIB + IC_80_ TC. Scales bar: 400 μm.

Some of the symptoms found in the previous measurements, such as the presence of adventitious roots, root hairs close to the meristem, or the altered cellular division pattern, suggested that auxin could play a role in the effect that TC has on treated roots. Therefore, a bioassay in the presence of the antiauxin PCIB was conducted to observe if TC effects could be reverted. As observed in **Figure 6**, control seedlings (**Figure 6A**) and PCIB-treated seedlings (15 and 30 µM PCIB, **Figures 6D, G**), just showed one main root with root hairs close to the transition zone, between the root and the hypocotyl. Moreover, as expected, seedlings treated with IC_50_ TC showed adventitious roots and short root hairs in non-common areas (**Figure 6B**), which were even more evident in those roots treated with IC_80_ (**Figure 6C**). However, when the seedlings were treated with the combined treatment PCIB/TC, the number of adventitious roots was reduced. However, hairs were still located in abnormal areas, and the root’s length was shorter than those of the control plants (**Figures 6E, F, H, I**), so PCIB only partially reverted TC effects.

**Figure 7:**
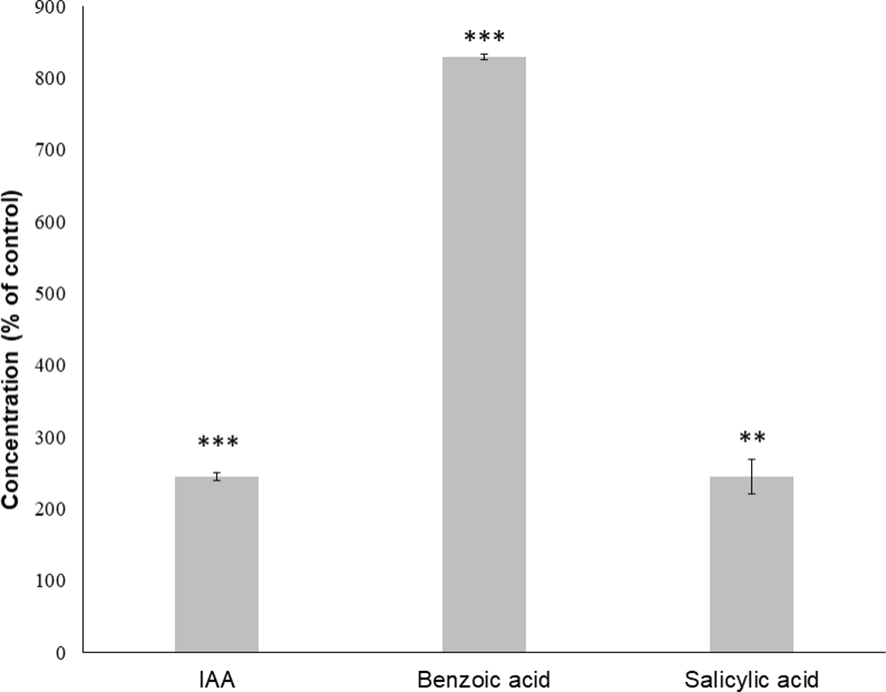
Relative concentration of *Arabidopsis* plant hormones identified through GC-MS after 14 days of treatment with the IC_50_ of TC. Asterisks indicate significant differences compared to the control: ** (*p* ≤ 0.01), *** (*p* ≤ 0.001); data were analysed through t-test with *p* ≤ 0.05. Bars indicate standard deviation.

GC-MS confirmed the role of auxin, showing that IAA content in seedlings treated with IC_50_ TC increased significantly (approximately by 200% compared to the control; **Figure 7**) after 14 days of treatment, although salicylic acid also registered a similar increase to IAA, the strongest increase was registered for benzoic acid, which surprisingly increased around 900% compared to the control.

**Figure 8:**
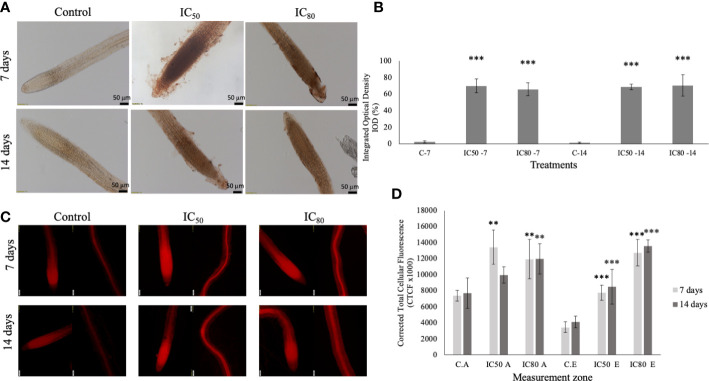
**(A)** *In situ* determination of H_2_O_2_ in *A*. *thaliana* seedlings control **(C)** and treated with the IC_50_ and IC_80_ of TC for 7 and 14 days. Scale: 50 μM; **(B)** quantification of the integrated optical density (IOD) expressed as a percentage of the root area stained with DAB. C-7 (control 7 days), IC_50_-7 (IC_50_ of TC after 7 days), IC_80_-7 (IC_80_ of TC after 7 days), C-14 (control 14 days), IC_50_-14 (IC_50_ of TC after 14 days), IC_80_-14 (IC_80_ of TC after 14 days). Asterisks indicate significant differences compared to the control: *** (*p* ≤ 0.001); **(C)** *in situ* determination of O_2_^−^ in *A*. *thaliana* seedlings treated with IC_50_ and IC_80_ TC for 7 and 14 days in apex and root elongation zones. Scale: 50 μm; **(D)** quantification of corrected total cellular fluorescence (CTCF) of roots stained with DHE. C.A (control apex), IC_50_ A (IC_50_ apex), IC_80_ A (IC_80_ apex), C.E (control elongation zone), IC_50_ E (IC_50_ elongation zone), IC_80_ E (IC_80_ elongation zone). Asterisks indicate significant differences compared to the control: ** (*p* ≤ 0.01), *** (*p* ≤ 0.001). Bars indicate standard deviation.

Regarding the determination of reactive oxygen species H_2_O_2_ and O_2_^−^, as shown in **Figure 8A**, the seedlings treated with IC_50_ and IC_80_ TC caused an increase in the H_2_O_2_ content, both at 7 and 14 days of treatment, being highly significant in all the cases, with DAB stained areas that oscillated between the 65 to 70% of the root (**Figure 8B**), while in control roots, the areas marked with the characteristic reddish brown of DAB staining did not exceed 3%. Regarding the presence of O_2_^−^, the seedlings treated with TC showed an increase in DHE fluorescence similar to H_2_O_2_, being much more significant in the elongation zone than in the root tips (**Figures 8C, D**). Only the IC_50_ TC-treated roots for 14 days did not show differences with respect to the control in terms of the presence of O_2_^−^ in the apices of the treated radicles, although those significant differences remained in the elongation zone.

In contrast to reactive oxygen species, MDA content did not show significant differences between control and IC_50_ TC-treated seedlings for 14 days (**Supporting information Figure S3**).

Based on the previous benzoic acid content results, *trans*-cinnamaldehyde oxidation was in silico tested by molecular docking studies on aldehyde dehydrogenase family 2 member B4 (ALDH2B4) of *A. thaliana.* To generate 3D models, the crystal structure of ZmALDH2 was found to be an appropriate template for model building since it has 58.0% identity.

The ICM-homology model showed a good plot of energies for each amino acid (**Supporting information Table 1**), using ICM Protein Health tool. Moreover, all residues, except Cys474 and Ser477, were within the allowed regions of the Ramachandran plot (**Supporting information Figure S4**).

**Figure 9:**
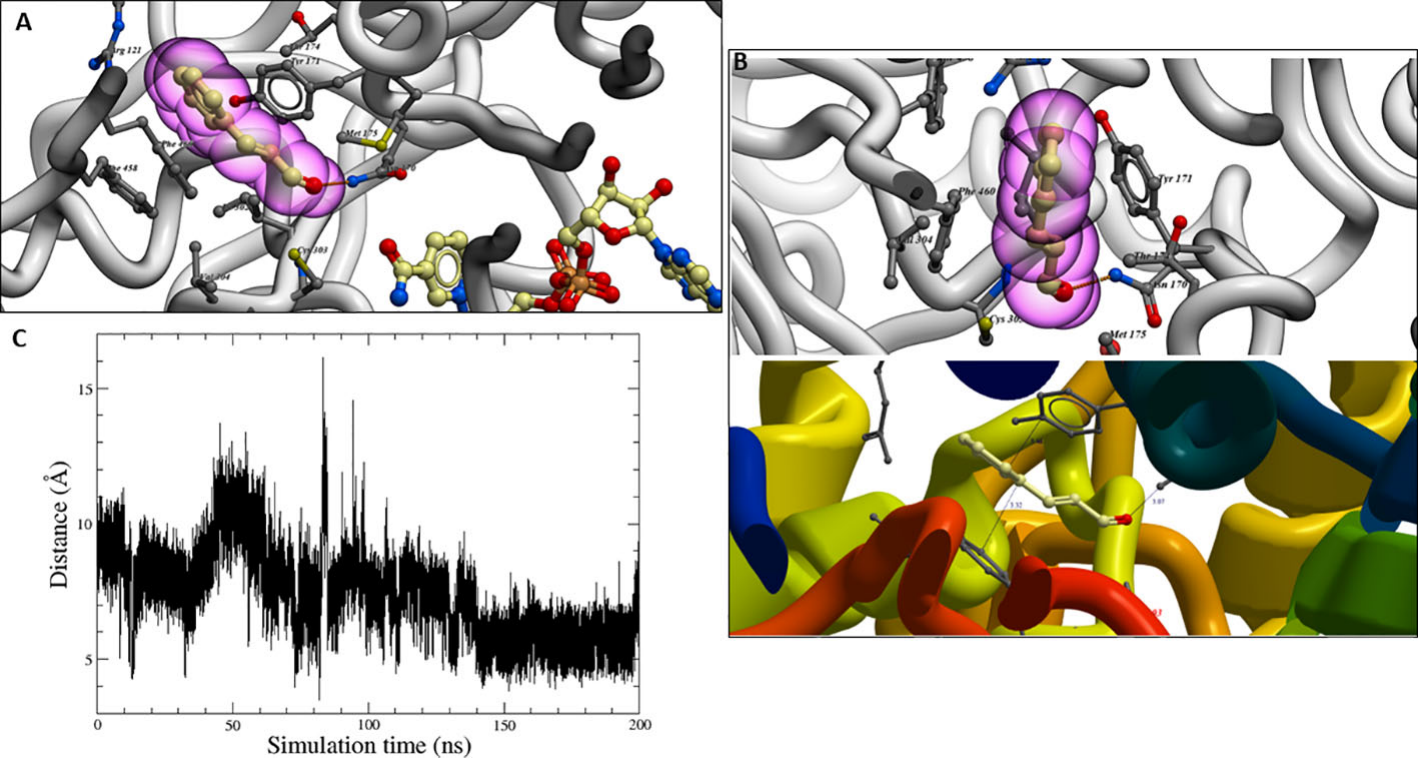
**(A)** *Trans*-cinnamaldehyde in the binding site of homology model (ALDH2). **(B)** Distances of π−π stacking and hydrogen-bond interactions in molecular docking studies. **(C)** Distance between the hydrogen HD1 in TC and the center of mass of Cys-303 throughout the MD simulation.

The best binding poses obtained after molecular docking calculations placed the *trans*-cinnamaldehyde on a hydrophobic pocket next to the NAD^+^ cofactor, as shown in **Figure 9A**, being available to be converted to cinnamic acid. Thus, the aldehyde group is located close to the conformational space of Cys-303. Particularly noteworthy is the strong hydrogen-bonding between the Asn-170 carboxamide group and the oxygen atom of *trans*-cinnamaldehyde (interactions N–H···O), which could stabilise the TC-ALDH2B4 complexes.

Two interesting π–π stacking interactions with two hydrophobic residues, Phe-460 and Tyr-171, in both sides of the aromatic ring could also be observed (**Figure 9B**). Since, in *trans*-cinnamaldehyde structure, a conjugate α, β-unsaturated aldehyde removes electron density from π system (stabilised by resonance, as shown in Supporting information, **Figure S5**), could be stabilised by both π-stacking interactions. This π–π stacking strengthens the ligand’s placement and avoids the aromatic ring’s rotation.

Molecular dynamic simulations were performed to obtain more comprehensive structural information about the stability of the complex TC-ALDH2B4. During molecular dynamics simulations, the TC ligand remained buried in the funnel-shaped cavity with an opening leading to the catalytic pocket, which harbours the important catalytic thiol, Cys-303 (**Figure 9C**).

Based on these molecular docking and molecular dynamic simulation results, the relative gene expression of the different *ALDH* genes in IC_50_ TC-treated roots and shoots was studied, finding clear differences in the expression patterns of these genes in roots or shoots between control and TC-treated seedlings.

**Figure 10:**
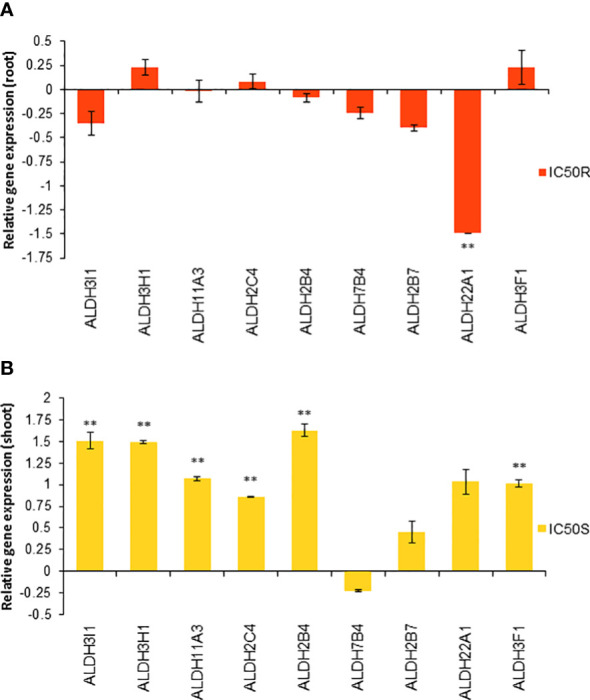
Relative gene expression of different *ALDH* genes in *Arabidopsis* seedlings treated with the IC_50_ TC in roots **(A)** and shoots **(B)** for 14 d. Asterisks indicate significant differences compared to the root control for treated roots and to the shoot controls for treated shoots: ** (*p* ≤ 0.05). Bars indicate standard error.

Although under control conditions the gene expression of most of the *ALDH* genes in roots was stronger than in shoots (**Figure S6**), no significant expression changes of the *ALDH* genes were observed in the TC-treated roots (**Figure 10A**), with the only exception of the *ALDH22A1* gene, which was significantly repressed, almost 1.5-fold less with respect to the control roots. However, a completely different situation was observed for the TC-treated shoots (**Figure 10B**), where most of the *ALDH* genes were overexpressed compared to the control, Except for *ALDH7B4*, *ALDH2B7* and *ALDH22A1* genes, whose expression was not altered by the IC_50_ *trans*-cinnamaldehyde treatment, the rest of the genes were significantly overexpressed compared to the control shoots. Some of them, such as *ALDH3I1*, *ALDH3H1* and *ALDH2B4*, showed about 1.5-fold overexpression compared to the control shoots.

## 4 Discussion

### 4.1 Morphological and physiological effects induced by *trans*-cinnamaldehyde

The results obtained show a strong effect of TC on the root morphology of *Arabidopsis* seedlings, with significant structural and ultrastructural alterations, such as the formation of adventitious roots, increasing number of lateral roots, and the occurrence of short and abundant root hairs, some of them behaving as ectopic root hairs. Many other plant secondary metabolites can alter the root architecture of treated-plants. For example, the indole alkaloid norharmane (López-González et al., 2020) caused the emergence of adventitious roots on *Arabidopsis thaliana* seedlings by altering the polar auxin transport (PAT), while scopoletin, a phenolic compound as TC, caused torsion effects in the roots as a consequence of alterations in the microtubules, resulting in more lateral roots in *A. thaliana* seedlings when competing with auxin for the TIR1 binding site (Graña et al., 2017).

The role played by auxin in the mode of action of TC was first demonstrated by the partial reversion of TC’s effect found in the PCIB reversion bioassay and then confirmed by GC-MS quantification of TC-treated seedlings, where auxin levels increased more than 200% when compared with untreated seedlings. In fact, the phenotype observed in TC-treated seedlings (inhibition of root length and appearance of adventitious roots) is very similar to that observed in the *Arabidopsis* mutant overproducing auxin superroot (Boerjan et al., 1995).

GC-MS analysis also showed a 200% increase in salicylic acid (SA) and a 900% increase in benzoic acid (BA). The synthesis of both compounds starts with the synthesis of *trans*-cinnamic acid from phenylalanine (Moerkercke et al., 2009). This cinnamic acid can be transformed by cinnamyl alcohol dehydrogenases (CAD) to produce monolignols, the fundamental units of lignin. The expression of the *CAD* genes, especially *CAD-C* and *CAD-D*, has important defensive functions in the plant (Tronchet et al., 2010; Proietti et al., 2021), so a higher production of cinnamic acid may be an indicator of a stress situation in the plant. Subsequently, *trans*-cinnamic acid can be transformed into BA via three different biosynthetic routes: a β-oxidative route, which requires CoA, a non-β-oxidative CoA-dependent pathway and a non-β-oxidative CoA-independent pathway (Widhalm and Dudareva, 2015). Benzoic acid is then transformed into SA through the benzoic-acid-2-hydroxylase (Shah, 2003). Araniti et al. (2018b) observed how the addition of *trans*-cinnamic acid to maize leaves led to an increase in the content of BA and SA.

### 4.2 Role of ALDHs TC-induced metabolic alterations in *Arabidopsis* seedlings

Based on our results with TC and the previous results with cinnamic acid, the presence of TC in the plant could increase the levels of BA and SA, which could be the result of the transformation of TC in cinnamic acid. This transformation can be performed by direct aerobic oxidation of cinnamaldehyde to cinnamic acid employing silica-supported Pt nanoparticles (Durndell et al., 2019), but little is known about its transformation inside the plant, which can occur through aldehyde-dehydrogenases enzymatic reactions. Aldehyde-dehydrogenases (ALDHs) are NAD(P)^+^ dependent enzymes that catalyse the irreversible oxidation of aldehydes to carboxylic acids. Molecular docking assays showed the strong affinity of TC for a hydrophobic pocket next to the NAD^+^ cofactor of the ALDH2B4 enzyme, which seems to be involved in the enzymatic reaction mechanism of ALDHs (Muñoz-Clares et al., 2017) and could transform TC into cinnamic acid, which would explain the high levels of BA and SA observed. These results are also supported by molecular dynamics (MD) simulations, which place the docking complex between the TC ligand on the target protein ALDH2B4 in the catalytic pocket throughout the simulation process.

When gene expression studies were done to confirm the molecular docking results, a differential expression of these genes was found depending on the part of the plant. Under control conditions, there were more genes expressed in roots than in shoots. However, after TC-treatment, the number of genes expressed in shoots strongly increased when compared to untreated shoots. On the contrary, the expression for the *ALDH* genes in TC-treated roots did not show relevant changes compared to untreated roots (**Figure 10**). The *Arabidopsis* genome contains 14 unique *ALDH* sequences encoding members of nine *ALDH* gene families, including one new family (ALDH22) that is currently known only from plants (Kirch et al., 2004). The aldehyde-dehydrogenase family 2 (ALDH2) could be involved in the oxidation of aromatic aldehydes, such as benzaldehyde, anisaldehyde, cinnamaldehyde, or coniferaldehyde (Končitíková et al., 2015). The three ALDHs belonging to family 2 (ALDH2C4, ALDH2B4 and ALDH2B7) are likely involved in the conversion of acetaldehyde into acetic acid, which can increase drought tolerance (Bashir et al., 2022). Moreover, the expression level of these genes was increased in these drought stress situations (Rasheed et al., 2018). ALDH2B4 is an enzyme involved in IAA synthesis (Hou et al., 2020; Zhang et al., 2022). It is possible that the observed increase in the expression of this enzyme is related to the increase in IAA detected by GC-MS. In other stressful situations, such as oxidative stress, there is an overexpression of *ALDH* genes belonging to families 3 and 7, particularly *ALDH3H1, ALDH3I1, ALDH3F1* and *ALDH7B4*, which encode enzymes capable of detoxifying aldehydes produced during lipid peroxidation (such as MDA) (Stiti et al., 2011). The expression level of the *ALDH3H1* gene, which is more expressed in roots (Kirch et al., 2005), was significantly increased both in roots and shoots, while *ALDH3I1* (typically expressed in shoots) and *ALDH3F1* gene expression levels were significantly increased only in shoots. As previously explained, the only down-regulated gene after TC-treatment was *ALDH22A1* in roots, which is in accordance with previous studies (Islam et al., 2022) that showed down-regulation of *ALDH22A1* in roots in response to stress.

Just the expression of the *ALDH7B4* gene was not altered at all after TC treatment. TC would only activate the expression of family 3 genes, while it would not activate the expression of family 7 genes. Finally, family 11 catalyses the NADP^+^ dependent oxidation of glyceraldehyde-3-phosphate to 3-phosphoglycerate and NADPH. This reaction is the main source of NADPH for plant mannitol synthesis (Brocker et al., 2013). Treatment with TC caused an increase in the expression of the *ALDH11A3* gene in shoots.

### 4.3 Increases in BA and SA related to oxidative stress in TC-treated seedlings

The overexpression of aldehyde-dehydrogenase (*ALDHs*) genes would be coherent with an increase of CA into the shoots and its transformation to BA and SA. Several studies have shown that BA is able to alter the ultrastructure of the roots, reducing the size of the meristematic zone and the number and shape of the cells, which causes also alterations to the mitochondria (Zhu et al., 2017; Zhang et al., 2018), which are like the alterations observed after treatment with TC. In this sense, it was observed that the exogenous application of BA caused the reduction of the main root due to auxin accumulation in the root apex via an increase in the auxin synthesis and transport through transporters AUX1 and PIN2, besides causing an increase in the production of reactive oxygen species (ROS) (Zhang et al., 2018). The high levels of BA observed in TC-treated seedlings could increase auxin levels and facilitate their transport. Several studies also showed how exogenous application of BA causes oxidative stress in different plant species that would end up triggering lipid peroxidation processes, as observed in an increase of MDA in treated plants (Baziramakenga et al., 1995; Yadav and Singh, 2013; Sunaina and Singh, 2015). However, TC did not cause an increase in lipid peroxidation, as shown by the MDA content, which could be due to the action of aldehyde dehydrogenases, which can detoxify peroxidative aldehydes such as MDA.

BA can also be used to produce SA (Ahmad et al., 2019), another compound that can be found at high levels in TC-treated seedlings. SA is a phytohormone that plays a key role in inducing tolerance against biotic and abiotic stress situations (Vlot et al., 2009; Khan et al., 2015). Low SA levels promote stress tolerance by inducing antioxidant responses, while high levels induce ROS overproduction and inhibit antioxidant enzymes (Durner and Klessig, 1996; Janda et al., 2003; Kawano, 2003). This behaviour is caused by a self-amplifying feedback loop between SA and H_2_O_2_, meaning that high levels of SA induce the production of H_2_O_2_ (Hara et al., 2012).

The increase in ROS, particularly superoxide anion (O_2_^−^) and hydrogen peroxide (H_2_O_2_) in TC-treated radicles, together with the effect of BA, could result in oxidative stress in TC-treated seedlings. Superoxide anion is generally in less quantity since it is normally transformed into hydrogen peroxide (Zarepour et al., 2012). This superoxide anion is more commonly observed in the elongation zone, while hydrogen peroxide is more commonly observed in the apices in the differentiation zone (Dunand et al., 2007), as found in TC-treated roots.

The phenotype with a shorter root length and an increase in root branching is typical when a plant is exposed to stress-inducing agents (Pasternak et al., 2005b). Pasternak et al. (2005a) observed that when *A. thaliana* seedlings were treated with two compounds that induce oxidative stress, alloxan and paraquat, the growth was reorientated. This reorientation was induced by an inhibition in the expression of auxin transporters PIN1 and PIN3 and an increase in the expression of Aux synthesis genes, which caused an irregular auxin distribution. As auxin is unevenly distributed, it accumulates in different areas, causing the activation of the cell division and greater branching of the root system, a phenotype similar to that found in TC-treated seedlings. Another example of the relationship between oxidative stress and alterations in auxin function was observed in the work of (Kim et al., 2021). They observed how methyl-bromide induces phytotoxic damage in *A. thaliana* seedlings, causing oxidative stress and altering auxin homeostasis through reduced expression of auxin transporter proteins, leading to alterations in root architecture. These examples show that compounds that cause oxidative stress can alter root growth and morphology by altering auxin homeostasis.

Oxidative stress also harms mitochondrial function (Sweetlove et al., 2002). The increase in ROS during oxidative stress causes the uncoupling of the electron transport chain of cellular respiration, which reduces ATP production (Tiwari et al., 2002). Therefore, cells tend to produce more mitochondria to compensate for the ATP deficit (Hardie, 2011), as found in cells of TC treated seedlings. One of the most common effects of ROS is the degradation of the membranes, which leads mitochondrial membranes to depolarise and become more permeable, thus enabling the release of mitochondrial molecules such as cytochrome c (Yao et al., 2004). The alteration in the polarisation of the mitochondrial membrane of those roots treated with TC was demonstrated in this research paper by using JC-1 as a dye, thus observing a membrane depolarisation in the mitochondria of treated cells.

Lastly, one of the most striking ultrastructural effects is the increased Golgi activity after 14 days TC treatment, with numerous small vesicles observed in the cytoplasm and a high number of different-sized vacuoles. These organelles take part in detoxification processes. In the presence of xenobiotics, the cells try to eliminate them through compartmentalisation in the vacuoles or deposition into the cell walls (Kaur et al., 2005).

## 5 Conclusion

In conclusion, TC could be converted to CA, as suggested by the overexpressed aldehyde dehydrogenases, which could result in the increased levels of IAA, BA and SA found after TC treatment. The increase of these molecules could be the cause of the alterations in growth pattern and oxidative stress in the treated seedlings, which could provoke a mitochondrial malfunction that would trigger PCD processes.

## Data availability statement

The original contributions presented in the study are included in the article/Supplementary Files, further inquiries can be directed to the corresponding author/s.

## Author contributions

Conceptualization, MJR and AS-M. Methodology, FA and AS-M. Software, DL-G, YF and JH-R. Validation, DL-G, YF, FA, EG, JH-R, MG, MT, MR, MJR and AS-M. Formal analysis, DL-G, YF, FA, EG, and JH-R. Experimental work, DL-G, YF, FA, EG, JH-R, MG, MT, MR, MJR and AS-F. Resources, MG, MT, MR, MJR and AS-M. Data curation, DL-G, YF, FA, JH-R and AS-M. Writing—original draft preparation, DL-G. Writing—review and editing, YF, FA, MG, MT, MR and AS-M. Visualization, AS-M. Supervision, MT, MR and AS-M. Project administration, AS-M. Funding acquisition, AS-M. All authors contributed to the article and approved the submitted version.
