# Supplementary material for: Trans-cinnamaldehyde-related overproduction of benzoic acid and oxidative stress on Arabidopsis thaliana
Source: Front Plant Sci. 2023 Apr 21;14:1157309. doi: 10.3389/fpls.2023.1157309 (PMC10160683; doi:10.3389/fpls.2023.1157309)
Supplement: Supplementary file 1 [file Table_1.docx]

Supplementary Material

*Trans*-cinnamaldehyde conversion to cinnamic acid in the plant causes overproduction of benzoic acid and oxidative stress on treated seedlings

David López-González^*^, Yolanda Ferradás, Fabrizio Araniti, Elisa Graña, José M. Hermida-Ramón, María Victoria González, Marta Teijeira, Manuel Rey, Manuel J. Reigosa, Adela M. Sánchez-Moreiras

*** Correspondence:** David López-González: davidlopez@uvigo.gal

# Supplementary Data

**Table S1.** Protein Health scores in the ICM homology model for Arabidopsis thaliana aldehyde dehydrogenase 2 (At-ALDH2B4).

| **Residue** | **Normalized Energy** | **Phi** | **Psi** |
| --- | --- | --- | --- |
| leu461 | 6.205496 | -57 | -33 |
| met470 | 5.240476 | -92 | 13 |
| asn169 | 4.970931 | -67 | -39 |
| leu269 | 4.849441 | -123 | 41 |
| tyr296 | 4.829914 | -58 | 132 |
| asp108 | 4.773975 | -79 | -51 |
| ile166 | 4.770914 | -119 | 145 |
| phe335 | 4.727094 | -78 | -23 |
| met236 | 4.392903 | -80 | -18 |
| his235 | 4.37648 | -69 | 135 |
| met1 | 4.293418 | 0 | 0 |
| met393 | 4.191203 | -72 | 151 |
| his72 | 4.101315 | -121 | 2 |
| gln196 | 4.09132 | -74 | -34 |
| lys298 | 4.055613 | 58 | 30 |
| cys302 | 4.003118 | -65 | -27 |
| leu267 | 3.938679 | -140 | 131 |
| trp329 | 3.886999 | -49 | 131 |
| asn297 | 3.81986 | 49 | 44 |
| met479 | 3.806268 | -72 | -26 |
| met189 | 3.804579 | -122 | 153 |
| arg307 | 3.724773 | -113 | 110 |
| met145 | 3.703349 | -78 | 144 |
| ile159 | 3.548005 | -70 | -45 |
| met183 | 3.479632 | -71 | -42 |
| thr20 | 3.409597 | -123 | 0 |
| ile450 | 3.346546 | -133 | 131 |
| leu150 | 3.317005 | -86 | 161 |
| leu291 | 3.286837 | -54 | -49 |
| met174 | 3.245995 | -64 | -38 |
| met405 | 3.238786 | -117 | 141 |
| asp280 | 3.224894 | -83 | -6 |
| leu262 | 3.190945 | 61 | 43 |
| glu96 | 3.1822 | -90 | -13 |
| lys469 | 3.140963 | 52 | -128 |
| ile121 | 3.048749 | -64 | -52 |
| cys474 | 3.029227 | 83 | 158 |
| asn484 | 2.963373 | -69 | -23 |
| thr244 | 2.940196 | -143 | 132 |
| trp75 | 2.899778 | -54 | -55 |
| gly45 | 2.887828 | 94 | 9 |
| glu476 | 2.855085 | -112 | 155 |
| lys263 | 2.843428 | -75 | 148 |
| leu322 | 2.839302 | -67 | -42 |
| lys192 | 2.834347 | -122 | 91 |
| cys187 | 2.789163 | -91 | 161 |
| glu268 | 2.767114 | -130 | 114 |
| glu363 | 2.750109 | -83 | -10 |
| lys240 | 2.7425 | -154 | 151 |
| ala119 | 2.720136 | -124 | -69 |
| gly364 | 2.704521 | 98 | 25 |
| trp168 | 2.693237 | -108 | -18 |
| val107 | 2.646442 | -68 | -29 |
| phe459 | 2.611964 | -72 | 147 |
| ala259 | 2.571163 | -93 | 2 |
| met408 | 2.561161 | -135 | 166 |
| gln397 | 2.549479 | -105 | -37 |
| ile436 | 2.503963 | -62 | -40 |
| val303 | 2.484783 | -108 | 13 |
| ile173 | 2.457475 | -62 | -49 |
| ser33 | 2.439567 | -77 | -26 |
| val492 | 2.40575 | -137 | 138 |
| leu432 | 2.385535 | -121 | 100 |
| val345 | 2.374803 | -59 | -45 |
| leu495 | 2.352513 | -124 | 152 |
| thr247 | 2.35073 | -60 | -48 |
| gly212 | 2.337286 | 112 | 14 |
| thr338 | 2.286984 | -84 | -18 |
| ile98 | 2.272856 | -59 | -45 |
| gln344 | 2.25968 | -68 | 162 |
| ile220 | 2.223534 | -125 | 121 |
| lys272 | 2.214457 | -144 | 73 |
| leu438 | 2.187379 | -60 | -45 |
| tyr485 | 2.179696 | -105 | 20 |
| ser198 | 2.17672 | -103 | -13 |
| asn421 | 2.154701 | -95 | 4 |
| asn261 | 2.15187 | -125 | 15 |
| phe81 | 2.149776 | -63 | -39 |
| asp346 | 2.146577 | -159 | 180 |
| phe401 | 2.145071 | 59 | 45 |
| ile213 | 2.130381 | -66 | 115 |
| leu407 | 2.11667 | -91 | 126 |
| thr146 | 2.041513 | -107 | -77 |
| thr222 | 2.028586 | -65 | 146 |
| ile40 | 2.015831 | -110 | 154 |
| ser260 | 2.012058 | -110 | -97 |
| thr384 | 2.011142 | -119 | 145 |
| ile301 | 2.008444 | -139 | 146 |
| leu14 | 1.987025 | -128 | 148 |
| lys111 | 1.968793 | -83 | 153 |
| asn422 | 1.949106 | -83 | 76 |
| glu195 | 1.938096 | -61 | -24 |
| tyr396 | 1.917526 | -97 | -15 |
| leu368 | 1.889105 | -90 | -44 |
| phe170 | 1.877774 | -138 | 79 |
| arg43 | 1.861481 | -79 | -39 |
| phe457 | 1.859339 | 46 | 47 |
| met78 | 1.855808 | -63 | 157 |
| val404 | 1.84964 | -144 | 121 |
| ser306 | 1.847561 | -118 | -44 |
| gly313 | 1.81035 | -60 | -36 |
| thr177 | 1.802607 | -52 | -32 |
| leu275 | 1.778043 | -131 | 114 |
| tyr315 | 1.777336 | -55 | -54 |
| val442 | 1.764446 | -66 | -46 |
| ile283 | 1.757218 | -60 | -53 |
| gly34 | 1.733286 | 87 | 7 |
| ala135 | 1.716622 | -61 | -35 |
| glu38 | 1.710789 | -108 | 142 |
| leu199 | 1.662098 | -68 | -61 |
| val217 | 1.659624 | -70 | -48 |
| glu300 | 1.656897 | -91 | 62 |
| val413 | 1.618884 | -70 | -44 |
| gln341 | 1.616222 | -146 | 127 |
| val249 | 1.615993 | -71 | -35 |
| trp500 | 1.612383 | -153 | 157 |
| ala180 | 1.603609 | -51 | -60 |
| ala9 | 1.596399 | -173 | 172 |
| asn164 | 1.59586 | -128 | 131 |
| ile253 | 1.588854 | -63 | -50 |
| his496 | 1.578999 | -136 | 132 |
| thr412 | 1.568506 | -105 | 162 |
| asn131 | 1.557241 | -73 | -32 |
| his127 | 1.556228 | -62 | -39 |
| leu292 | 1.55071 | -60 | -46 |
| lys377 | 1.54679 | -127 | 163 |
| tyr468 | 1.544849 | -114 | 167 |
| ser266 | 1.537046 | -137 | 136 |
| lys144 | 1.506709 | -117 | 87 |
| phe28 | 1.496752 | -88 | 113 |
| lys137 | 1.48956 | -129 | 19 |
| ile138 | 1.478869 | -74 | 124 |
| phe295 | 1.473896 | -107 | 5 |
| phe151 | 1.466274 | -85 | 143 |
| val12 | 1.461201 | -159 | 141 |
| leu143 | 1.458599 | -87 | 169 |
| thr227 | 1.456176 | -100 | -79 |
| lys178 | 1.452574 | -94 | -48 |
| lys372 | 1.43458 | -162 | 158 |
| cys6 | 1.432072 | -172 | 171 |
| ser477 | 1.429104 | 65 | 165 |
| ile17 | 1.426112 | -158 | 145 |
| asp120 | 1.422011 | -46 | -65 |
| ser273 | 1.420006 | -72 | 128 |
| ala134 | 1.413877 | -92 | -18 |
| asp392 | 1.400717 | -95 | -4 |
| met254 | 1.398644 | -61 | -41 |
| ile395 | 1.396432 | -73 | -14 |
| asn279 | 1.376982 | -62 | -32 |
| tyr204 | 1.364344 | -65 | -31 |
| ile314 | 1.363236 | -123 | 8 |
| gly186 | 1.324593 | 104 | 17 |
| asp41 | 1.314796 | -87 | 127 |
| tyr118 | 1.304128 | -78 | -44 |
| tyr356 | 1.283931 | -72 | -31 |
| phe90 | 1.276985 | -59 | -41 |
| pro15 | 1.276035 | -67 | 147 |
| gly140 | 1.274937 | -97 | -155 |
| glu100 | 1.260922 | -62 | -41 |
| his359 | 1.256973 | -56 | -45 |
| lys209 | 1.250181 | -63 | -42 |
| ile374 | 1.23282 | -142 | 164 |
| thr369 | 1.221017 | -159 | 160 |
| thr39 | 1.213293 | -109 | 143 |
| tyr456 | 1.207108 | -157 | 165 |
| leu86 | 1.194032 | -74 | -39 |
| tyr465 | 1.187677 | -139 | 120 |
| pro499 | 1.1819 | -69 | -18 |
| phe278 | 1.171513 | -93 | 170 |
| gly245 | 1.16773 | 168 | -138 |
| cys463 | 1.166692 | -98 | 120 |
| ile451 | 1.157312 | -117 | 123 |
| ile439 | 1.149139 | -59 | -48 |
| his139 | 1.133068 | -125 | 137 |
| val319 | 1.11171 | -48 | -53 |
| phe128 | 1.111138 | -68 | -36 |
| asn44 | 1.109071 | -119 | -34 |
| asp105 | 1.10494 | -62 | -30 |
| pro167 | 1.101941 | -69 | -168 |
| thr197 | 1.098635 | -137 | 80 |
| thr124 | 1.095874 | -68 | -45 |
| glu53 | 1.088812 | -99 | 100 |
| gly26 | 1.076739 | 69 | 21 |
| ile446 | 1.075978 | -76 | 130 |
| ile400 | 1.07584 | -90 | -38 |
| lys361 | 1.074728 | -65 | -45 |
| val265 | 1.065353 | -121 | 158 |
| gly73 | 1.060988 | -89 | 159 |
| gly4 | 1.057822 | 178 | 177 |
| cys455 | 1.040795 | -168 | 175 |
| leu354 | 1.04049 | -59 | -38 |
| phe203 | 1.033054 | -62 | -43 |
| ser200 | 1.014093 | -59 | -35 |
| ser149 | 1.005001 | 58 | 34 |
| gly109 | 0.994445 | -112 | -6 |
| leu61 | 0.994306 | -67 | -39 |
| ala258 | 0.985591 | -61 | -36 |
| thr79 | 0.972975 | -75 | 158 |
| gly472 | 0.965455 | 117 | -158 |
| gly449 | 0.953728 | -73 | -32 |
| gly299 | 0.93977 | 68 | 13 |
| phe224 | 0.937487 | -84 | 167 |
| thr10 | 0.93687 | -167 | 176 |
| val453 | 0.934354 | -104 | 124 |
| leu289 | 0.928298 | -70 | -42 |
| phe70 | 0.926951 | -85 | -50 |
| lys21 | 0.926021 | -99 | 174 |
| leu104 | 0.916304 | -63 | -49 |
| asn454 | 0.909012 | 56 | 32 |
| gln487 | 0.905834 | -89 | 151 |
| ser471 | 0.873536 | -82 | -16 |
| ile48 | 0.868861 | -84 | -58 |
| gln434 | 0.839995 | -104 | -18 |
| lys409 | 0.821261 | -117 | 149 |
| asp55 | 0.810214 | -127 | -167 |
| thr11 | 0.80642 | -169 | 164 |
| gly8 | 0.806204 | 178 | 177 |
| val59 | 0.793838 | -61 | -41 |
| ile417 | 0.783383 | -62 | -44 |
| ala205 | 0.781137 | -60 | -42 |
| his206 | 0.767316 | -68 | -45 |
| val63 | 0.76628 | -65 | -38 |
| val323 | 0.756092 | -60 | -48 |
| ser406 | 0.75418 | -114 | 108 |
| ala123 | 0.747279 | -70 | -29 |
| leu155 | 0.745526 | -114 | 142 |
| asn97 | 0.741107 | -111 | 30 |
| ile357 | 0.739974 | -61 | -44 |
| lys321 | 0.717843 | -69 | -36 |
| gln349 | 0.717488 | -67 | -40 |
| glu210 | 0.710041 | -61 | -36 |
| met493 | 0.700698 | -148 | 150 |
| asn3 | 0.698257 | -175 | 174 |
| lys156 | 0.696973 | -106 | 105 |
| ile51 | 0.681718 | -107 | 143 |
| ser498 | 0.641995 | -89 | 125 |
| gly216 | 0.638181 | 98 | -15 |
| ala281 | 0.637412 | -72 | 153 |
| phe380 | 0.636111 | -88 | 132 |
| asp60 | 0.635176 | -64 | -39 |
| ile232 | 0.632771 | -69 | -42 |
| gly402 | 0.625914 | -149 | 175 |
| asp333 | 0.617323 | -55 | 125 |
| val318 | 0.617053 | -67 | -46 |
| asp136 | 0.61702 | -101 | 18 |
| lys411 | 0.616713 | -110 | -60 |
| glu399 | 0.610601 | -77 | 117 |
| ala132 | 0.598989 | -58 | -45 |
| glu312 | 0.593533 | -59 | -28 |
| gly467 | 0.580373 | -98 | 163 |
| arg67 | 0.570976 | -62 | -46 |
| val241 | 0.56508 | -132 | 144 |
| ala481 | 0.561139 | -59 | -45 |
| ala373 | 0.551709 | -77 | 160 |
| ala201 | 0.541754 | -59 | -35 |
| asp58 | 0.539714 | -75 | -35 |
| arg83 | 0.53929 | -70 | -39 |
| val47 | 0.52468 | -71 | 133 |
| val179 | 0.522446 | -71 | -46 |
| phe19 | 0.516722 | -135 | 112 |
| asp435 | 0.508878 | -81 | 125 |
| ala230 | 0.508425 | -69 | -34 |
| asp237 | 0.501466 | -114 | 17 |
| lys56 | 0.494425 | -55 | -41 |
| ser305 | 0.492978 | -71 | 119 |
| tyr130 | 0.490468 | -59 | -49 |
| pro494 | 0.485613 | -69 | 162 |
| asn25 | 0.482077 | 59 | 40 |
| asp480 | 0.480013 | -59 | -38 |
| val238 | 0.434169 | -104 | 129 |
| ala233 | 0.430043 | -62 | -34 |
| ala339 | 0.425572 | -80 | 140 |
| glu414 | 0.417831 | -58 | -39 |
| asp71 | 0.416422 | -65 | -50 |
| gly160 | 0.409769 | 138 | -117 |
| lys347 | 0.406606 | -67 | -41 |
| gly225 | 0.386389 | -73 | -50 |
| phe309 | 0.384042 | -110 | 119 |
| ala69 | 0.372394 | -67 | -29 |
| gly133 | 0.363636 | -71 | -17 |
| gly110 | 0.363424 | 71 | 29 |
| tyr379 | 0.361752 | -98 | 58 |
| phe37 | 0.355793 | -126 | 160 |
| ala176 | 0.355239 | -72 | -35 |
| ile165 | 0.350612 | -126 | 120 |
| gly458 | 0.34912 | -88 | 54 |
| ile29 | 0.33736 | -133 | 158 |
| thr154 | 0.332397 | -113 | 146 |
| leu22 | 0.326631 | -76 | 151 |
| gly371 | 0.324738 | 84 | 29 |
| ser246 | 0.323565 | -81 | 158 |
| thr390 | 0.314728 | -93 | 160 |
| glu391 | 0.295436 | -67 | -22 |
| leu115 | 0.29343 | -67 | -35 |
| lys264 | 0.288422 | -67 | 144 |
| ser208 | 0.282784 | -60 | -47 |
| ile94 | 0.278559 | -57 | -41 |
| ala387 | 0.263008 | -112 | 174 |
| ala211 | 0.259123 | -69 | -27 |
| arg77 | 0.257964 | -82 | -17 |
| tyr425 | 0.25762 | -97 | 176 |
| cys294 | 0.246334 | -87 | -50 |
| phe23 | 0.227919 | -115 | 102 |
| pro74 | 0.225592 | -69 | -28 |
| ala420 | 0.222701 | -61 | -38 |
| ser226 | 0.20216 | -66 | -19 |
| ile24 | 0.19537 | -129 | 132 |
| leu93 | 0.192002 | -64 | -43 |
| asn497 | 0.189288 | 52 | 54 |
| lys117 | 0.184396 | -70 | -39 |
| asp388 | 0.16918 | 56 | 45 |
| thr423 | 0.160206 | -168 | 159 |
| ala32 | 0.157387 | -56 | -39 |
| thr488 | 0.156176 | -107 | 116 |
| leu112 | 0.148131 | -64 | 127 |
| asn88 | 0.145212 | -67 | -39 |
| gly466 | 0.12856 | -169 | 160 |
| lys35 | 0.112922 | -78 | 155 |
| ala185 | 0.11201 | -65 | -26 |
| glu157 | 0.104021 | -120 | 153 |
| ala231 | 0.103077 | -65 | -39 |
| pro122 | 0.101323 | -69 | -23 |
| val162 | 0.101175 | -124 | 138 |
| asp462 | 0.099911 | -92 | -12 |
| ser172 | 0.090623 | -68 | -52 |
| cys419 | 0.089168 | -64 | -42 |
| asp215 | 0.086061 | -58 | 138 |
| val331 | 0.076764 | -105 | 131 |
| pro464 | 0.049127 | -69 | 146 |
| gly332 | 0.046252 | 167 | -159 |
| lys89 | 0.046091 | -71 | -39 |
| gly430 | 0.029124 | -119 | 145 |
| asn219 | 0.02537 | -118 | 141 |
| glu99 | 0.024862 | -64 | -50 |
| ser445 | 0.023379 | -90 | -28 |
| phe350 | 0.021065 | -56 | -48 |
| ala49 | 0.018687 | -159 | 169 |
| leu218 | 0.017274 | -121 | 110 |
| gln382 | 0.016039 | -66 | 136 |
| arg129 | 0.013091 | -70 | -37 |
| ile431 | 0.012981 | -129 | 134 |
| leu202 | 0.012372 | -77 | -37 |
| ser234 | 0.008615 | -100 | 1 |
| val191 | 0.003219 | -102 | 138 |
| gly271 | -0.008657 | -178 | -165 |
| gly342 | -0.009073 | -98 | 179 |
| ala304 | -0.015242 | -59 | 137 |
| trp452 | -0.016033 | -109 | 116 |
| glu415 | -0.016499 | -62 | -46 |
| val491 | -0.027956 | -121 | 134 |
| thr188 | -0.028408 | -113 | 132 |
| ser337 | -0.041698 | -69 | -18 |
| phe175 | -0.050468 | -56 | -51 |
| leu427 | -0.055392 | -92 | -60 |
| phe243 | -0.058042 | -127 | 148 |
| phe386 | -0.071045 | -115 | 131 |
| leu276 | -0.072463 | -107 | 121 |
| lys489 | -0.080902 | -113 | 119 |
| ser443 | -0.08381 | -57 | -40 |
| arg251 | -0.113164 | -67 | -39 |
| leu367 | -0.12156 | -84 | 117 |
| ile381 | -0.138339 | -123 | 131 |
| gly116 | -0.139229 | -56 | -52 |
| thr142 | -0.139407 | -100 | 125 |
| ile87 | -0.151102 | -72 | -29 |
| ala31 | -0.151126 | -64 | 151 |
| gln114 | -0.1579 | -65 | -41 |
| pro76 | -0.170438 | -69 | -22 |
| asn7 | -0.178324 | -174 | 173 |
| ala228 | -0.179198 | -68 | -44 |
| ala256 | -0.184432 | -62 | -40 |
| asp398 | -0.185903 | -96 | 144 |
| ala106 | -0.188485 | -67 | -55 |
| tyr68 | -0.217192 | -66 | -37 |
| glu95 | -0.241023 | -65 | -29 |
| leu482 | -0.241384 | -62 | -41 |
| ala448 | -0.263891 | -140 | 154 |
| ala84 | -0.266061 | -61 | -43 |
| ala182 | -0.267892 | -60 | -44 |
| pro214 | -0.292707 | -69 | 160 |
| lys325 | -0.295996 | -66 | -42 |
| ala194 | -0.297873 | -63 | 144 |
| glu82 | -0.303931 | -69 | -33 |
| asn473 | -0.305476 | -153 | 159 |
| pro171 | -0.316899 | -69 | -38 |
| lys13 | -0.317366 | -93 | 128 |
| lys18 | -0.321563 | -156 | 164 |
| val389 | -0.32836 | -92 | 134 |
| glu324 | -0.336447 | -52 | -52 |
| ser242 | -0.336983 | -129 | 130 |
| gln148 | -0.338202 | -101 | -4 |
| lys252 | -0.33995 | -68 | -37 |
| ala365 | -0.343622 | -77 | 154 |
| pro274 | -0.346161 | -69 | 156 |
| gln311 | -0.346476 | -65 | 146 |
| lys424 | -0.349294 | -75 | -27 |
| ala326 | -0.373781 | -64 | -40 |
| phe113 | -0.392935 | -66 | -41 |
| ile277 | -0.410838 | -121 | 119 |
| met501 | -0.432342 | -142 | 154 |
| lys5 | -0.443435 | -171 | 174 |
| lys327 | -0.44459 | -75 | -32 |
| asp30 | -0.46835 | -81 | 167 |
| gly375 | -0.475572 | 88 | 164 |
| gly370 | -0.480673 | 68 | -140 |
| thr330 | -0.48789 | -87 | 115 |
| tyr153 | -0.494823 | -158 | 166 |
| gly293 | -0.496742 | -61 | -32 |
| glu46 | -0.512667 | -84 | 154 |
| ile353 | -0.541899 | -66 | -43 |
| asp460 | -0.556577 | -152 | 149 |
| asn440 | -0.584784 | -64 | -46 |
| lys447 | -0.586541 | -91 | 72 |
| asp239 | -0.601108 | -90 | -25 |
| leu207 | -0.604326 | -65 | -34 |
| ala286 | -0.616284 | -62 | -39 |
| ile385 | -0.619243 | -121 | 133 |
| ala125 | -0.623499 | -60 | -40 |
| thr36 | -0.626706 | -134 | 152 |
| asp288 | -0.633107 | -64 | -35 |
| ala428 | -0.639481 | -146 | 164 |
| lys352 | -0.642608 | -58 | -45 |
| ser433 | -0.645024 | -162 | 159 |
| ala429 | -0.647561 | -157 | 153 |
| gly163 | -0.65624 | -107 | 140 |
| val190 | -0.664286 | -121 | 117 |
| asp336 | -0.671079 | -79 | 132 |
| lys285 | -0.684091 | -62 | -50 |
| ala257 | -0.705253 | -63 | -47 |
| gly152 | -0.705526 | -148 | 158 |
| pro158 | -0.725497 | -69 | 145 |
| val221 | -0.738144 | -120 | 93 |
| phe410 | -0.743404 | -146 | 163 |
| gly229 | -0.749909 | -60 | -46 |
| leu486 | -0.759835 | -124 | 133 |
| lys418 | -0.760071 | -66 | -44 |
| ala65 | -0.762959 | -68 | -42 |
| lys317 | -0.779116 | -65 | -41 |
| thr50 | -0.782183 | -104 | 122 |
| gly378 | -0.785327 | 93 | 164 |
| asp92 | -0.798256 | -63 | -39 |
| ala184 | -0.828988 | -61 | -38 |
| gly250 | -0.850556 | -54 | -38 |
| gly426 | -0.876399 | -173 | 63 |
| asp284 | -0.877876 | -60 | -45 |
| ala287 | -0.895743 | -62 | -52 |
| lys103 | -0.905681 | -58 | -44 |
| lys85 | -0.905871 | -59 | -38 |
| glu57 | -0.912409 | -60 | -43 |
| gly126 | -0.927968 | -60 | -44 |
| val310 | -0.929447 | -114 | 135 |
| glu320 | -0.931616 | -58 | -46 |
| pro42 | -0.940328 | -69 | -12 |
| gly416 | -0.950889 | -68 | -35 |
| leu101 | -0.970682 | -63 | -47 |
| asp328 | -0.975835 | -82 | -20 |
| ala62 | -0.999484 | -65 | -46 |
| asp282 | -1.024229 | -74 | 122 |
| ser490 | -1.049078 | -92 | 125 |
| pro334 | -1.050489 | -69 | -12 |
| glu358 | -1.052104 | -64 | -45 |
| ala290 | -1.084102 | -67 | -43 |
| pro383 | -1.084377 | -69 | 126 |
| ala91 | -1.091738 | -64 | -44 |
| ala52 | -1.107846 | -60 | 138 |
| val161 | -1.152607 | -90 | 126 |
| asp483 | -1.154594 | -64 | -27 |
| asp316 | -1.169578 | -62 | -42 |
| gly54 | -1.199123 | -74 | 140 |
| thr441 | -1.216591 | -61 | -53 |
| pro403 | -1.245836 | -69 | 79 |
| glu351 | -1.257099 | -69 | -37 |
| asp437 | -1.277771 | -68 | -46 |
| pro181 | -1.28053 | -69 | -35 |
| lys394 | -1.287079 | -64 | -39 |
| val308 | -1.319059 | -104 | 122 |
| glu2 | -1.350297 | -170 | 171 |
| arg147 | -1.358815 | -64 | -41 |
| ala102 | -1.373304 | -59 | -43 |
| arg340 | -1.380439 | -110 | -14 |
| asp248 | -1.41257 | -53 | -50 |
| gly360 | -1.412699 | -51 | -48 |
| asn362 | -1.440008 | -65 | -31 |
| gly270 | -1.462312 | 79 | -168 |
| glu141 | -1.513026 | -133 | 147 |
| gly80 | -1.530546 | -57 | -41 |
| gln27 | -1.548262 | -137 | 143 |
| thr366 | -1.589442 | -98 | 125 |
| asp376 | -1.697976 | -80 | -52 |
| pro193 | -1.759643 | -69 | 155 |
| gln255 | -1.813412 | -65 | -42 |
| arg444 | -1.928101 | -77 | -24 |
| ser355 | -1.941832 | -63 | -46 |
| arg475 | -2.009356 | -121 | 138 |
| gly478 | -2.107476 | 77 | -180 |
| ala66 | -2.226295 | -65 | -40 |
| glu16 | -2.236973 | -147 | 120 |
| asn64 | -2.248416 | -62 | -40 |
| arg348 | -2.432952 | -55 | -51 |
| gly223 | -2.434656 | 158 | -177 |
| pro343 | -2.499734 | -69 | 163 |
