# Supplementary figures and images for: Trans-cinnamaldehyde-related overproduction of benzoic acid and oxidative stress on Arabidopsis thaliana
Source: Front Plant Sci. 2023 Apr 21;14:1157309. doi: 10.3389/fpls.2023.1157309 (PMC10160683; doi:10.3389/fpls.2023.1157309)

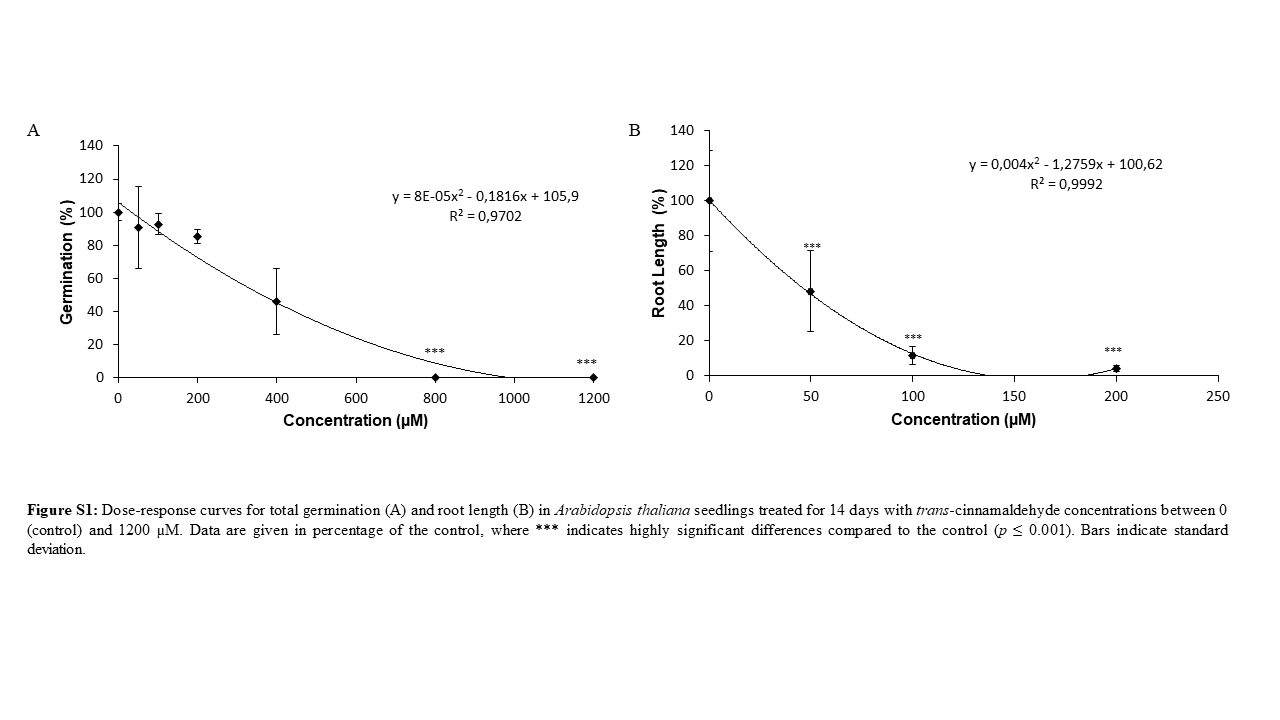

Supplement: Supplementary file 2 [file Image_1.tif]

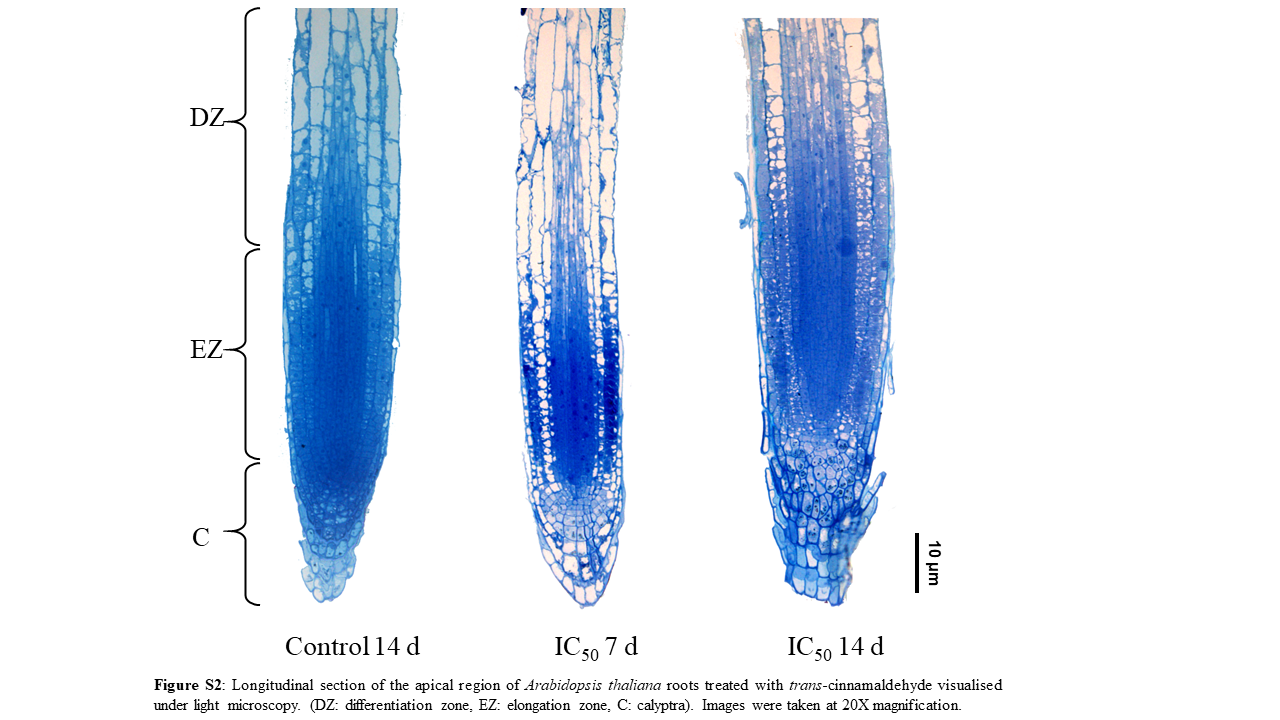

Supplement: Supplementary file 3 [file Image_2.tif]

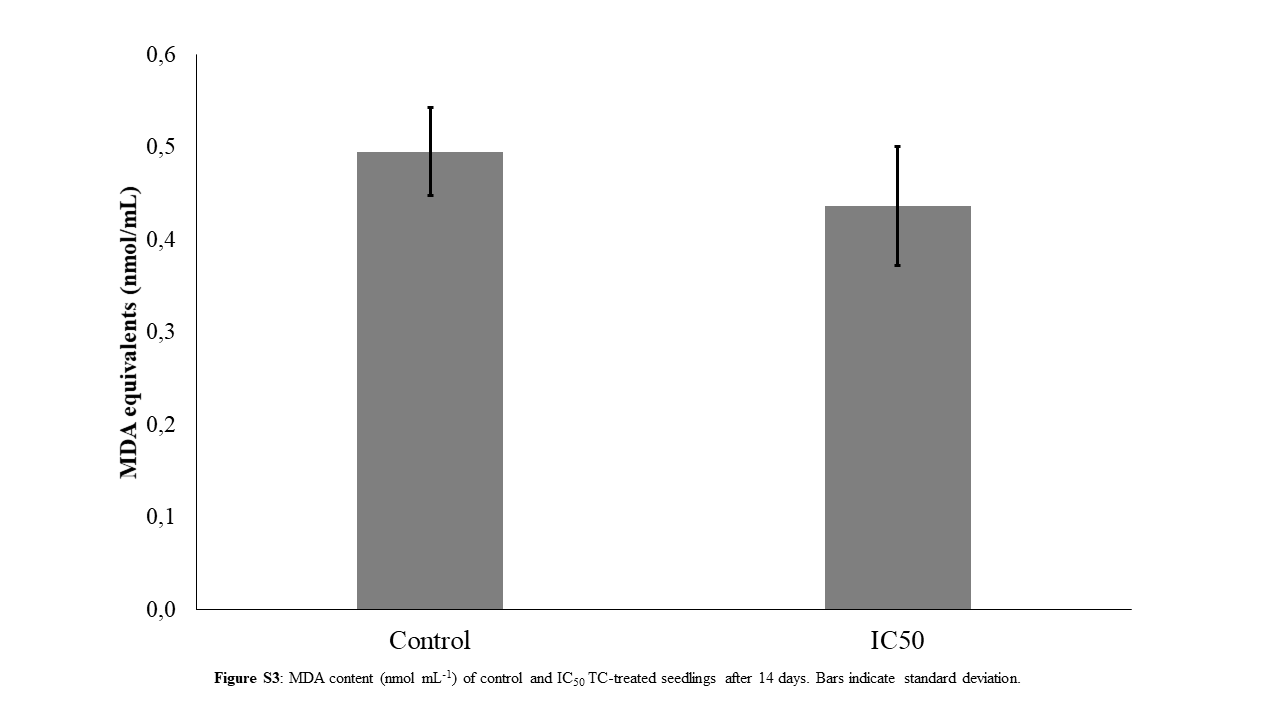

Supplement: Supplementary file 4 [file Image_3.tif]

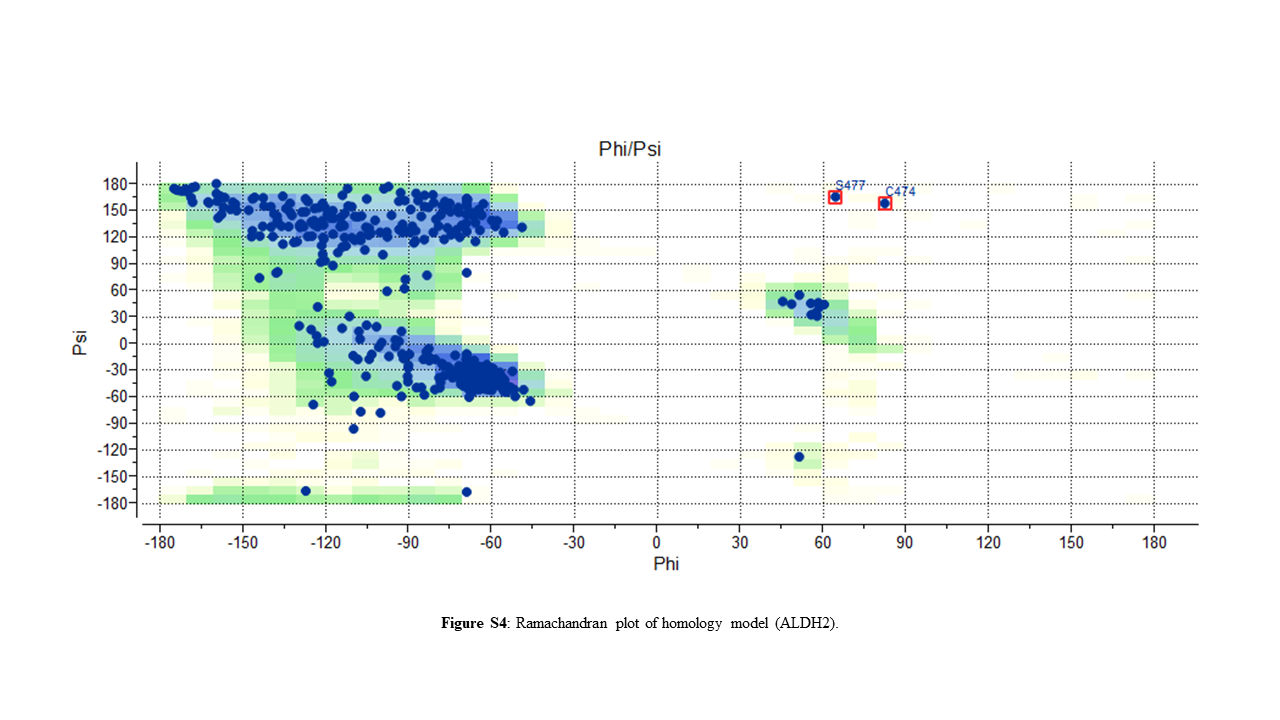

Supplement: Supplementary file 5 [file Image_4.tif]

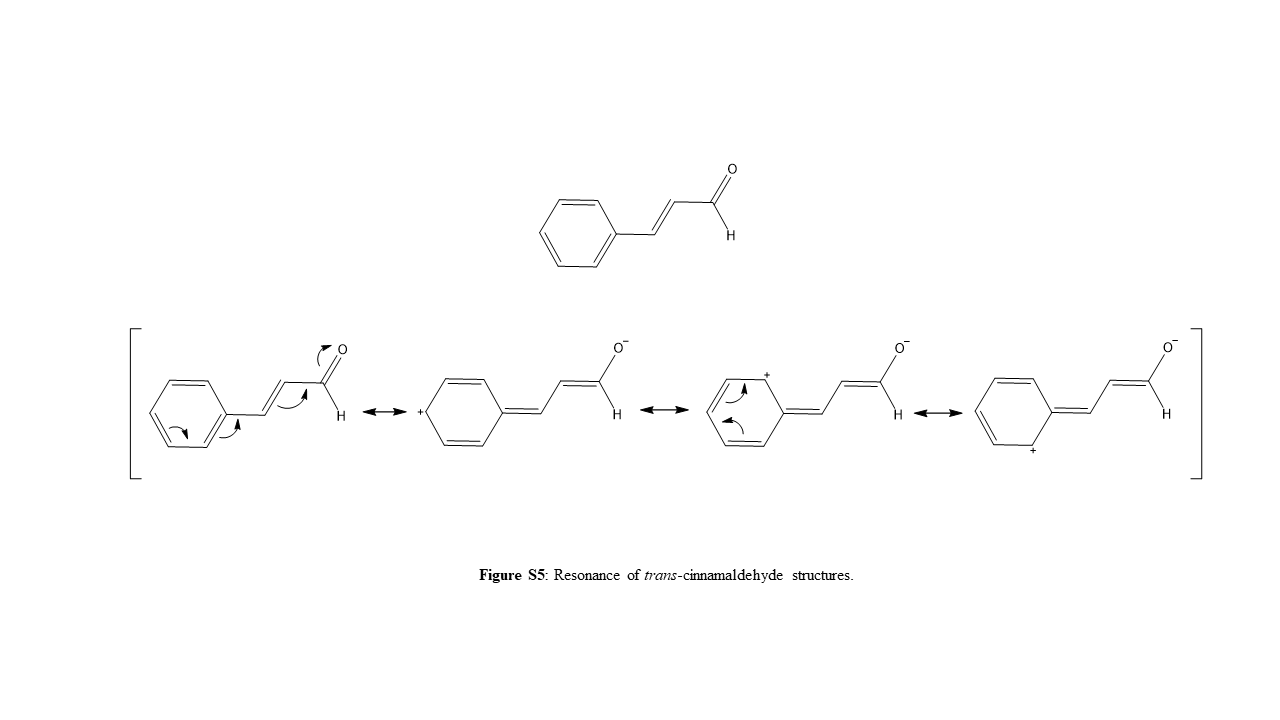

Supplement: Supplementary file 6 [file Image_5.tif]

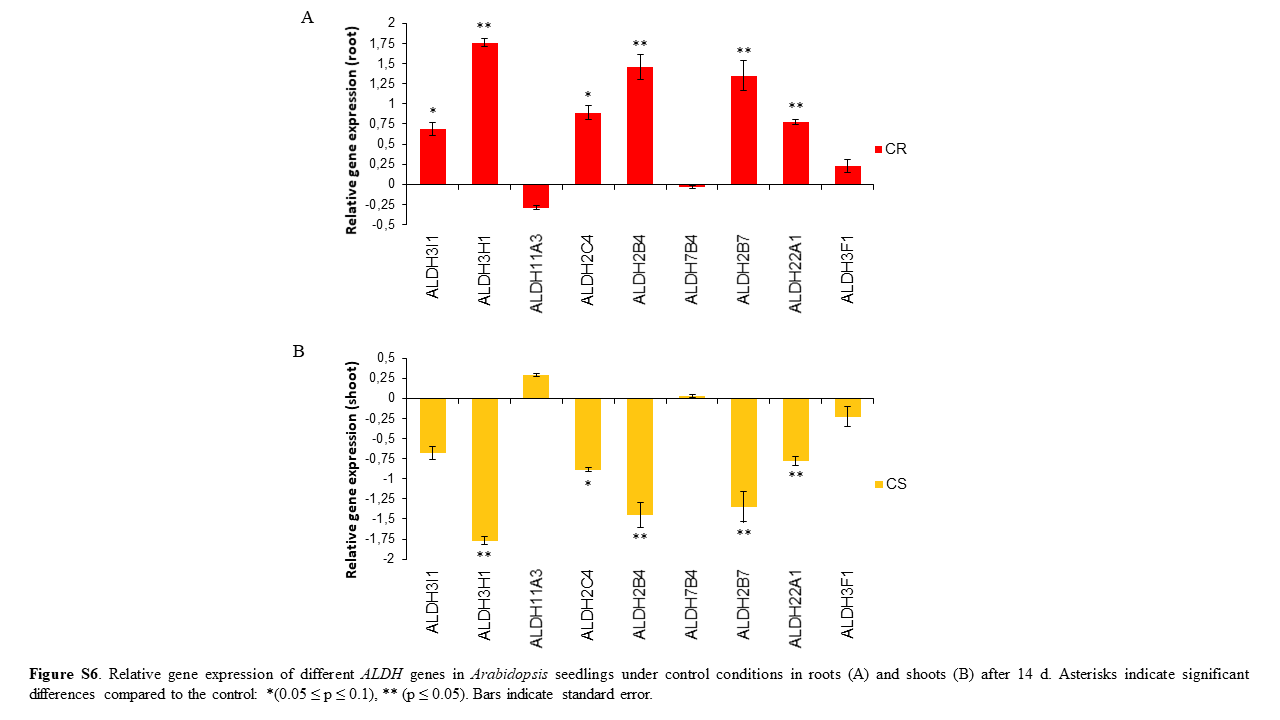

Supplement: Supplementary file 7 [file Image_6.tif]
